# The prevalence of comorbid serious mental illnesses and substance use disorders in prison populations: a systematic review and meta-analysis

**DOI:** 10.1016/S2468-2667(22)00093-7

**Published:** 2022-06-01

**Authors:** Gergő Baranyi, Seena Fazel, Sabine Delhey Langerfeldt, Adrian P Mundt

**Affiliations:** a Centre for Research on Environment, Society and Health, Department of Geography, School of GeoSciences, The University of Edinburgh, Edinburgh, UK; b Department of Psychiatry, University of Oxford, Oxford, UK; c Facultad de Medicina, Universidad Diego Portales, Santiago, Chile; d Department of Psychiatry and Mental Health, Medical Faculty, Hospital Clínico Universidad de Chile, Santiago, Chile; e Departamento de Neurología y Psiquiatría, Clínica Alemana de Santiago, Facultad de Medicina Clínica Alemana, Universidad del Desarrollo, Santiago, Chile

## Abstract

**Background:**

Comorbid mental illnesses and substance use disorders are associated with adverse criminal, social, and health outcomes. Yet, their burden is not reliably known among prison populations. We therefore aimed to estimate the prevalence of comorbid serious mental illnesses and substance use disorders (dual disorders) among people in prison worldwide.

**Methods:**

In this systematic review and meta-analysis, we searched 15 electronic databases (ASSIA, CAB Abstracts, Criminal Justice Database, Embase, Global Health, Global Index Medicus, IBSS, MEDLINE, NCJRS, PAIS Index, PsycINFO, Russian Science Citation Index, Scielo, Social Services Abstracts, and Web of Science) and the grey literature (Open Grey and ProQuest Dissertations & Theses Global) for studies reporting the prevalence of serious mental illnesses and substance use disorders in prison populations published between Jan 1, 1980, and Sept 25, 2021, and contacted the authors of relevant studies. Empirical studies among unselected adult prison populations that applied representative sampling strategies and validated diagnostic instruments, and either reported the prevalence of dual disorders or had authors who could provide prevalence data in correspondence, were included. Two reviewers (GB and SDL) independently extracted data from the eligible studies; both current (up to 1 year) and lifetime prevalence were extracted, if available. We sought summary estimates. Our primary outcomes were comorbid non-affective psychosis with substance use disorders and comorbid major depression with substance use disorders. We conducted a random-effects meta-analysis, explored between-sample heterogeneity with meta-regression, and calculated odds ratios (ORs) to assess bidirectional relationships between mental and substance use disorders. Risk of bias was assessed by use of a standard tool. The study protocol was registered with PROSPERO, CRD42020207301.

**Findings:**

Of 11 346 records screened, we identified 34 studies reporting the prevalence of dual disorders among individuals in prison and received unpublished prevalence data for 16 studies, totalling 50 eligible studies and 24 915 people. The mean quality score of included studies was 7·8 (SD 1·2). We found that 3·5% (95% CI 2·2–5·0) had current non-affective psychosis with any comorbid substance use disorder, representing 443 (49·2%) of 900 people with non-affective psychosis, and 9·1% (5·6–13·3) had current major depression and comorbid substance use disorders, representing 1105 (51·6%) of 2143 people with major depression. Between-sample heterogeneity was high (*I*^2^>80%). People in prison with current non-affective psychosis were significantly more likely to have substance use disorders compared with those without (OR 1·7, 95% CI 1·4–2·2). People with major depression had higher odds of substance use disorders than those without (1·6, 1·3–2·0).

**Interpretation:**

Around half of the prison population with non-affective psychosis or major depression have a comorbid substance use disorder. Consideration should be given to screening for dual disorders and implementing integrated and scalable treatments.

**Funding:**

Economic and Social Research Council, Agencia Nacional de Investigación y Desarrollo (Chile), and the Wellcome Trust.

## Introduction

In 2021, more than 11 million individuals were held in prisons around the world,^1^ a large proportion of whom had multiple mental health conditions.^2^ Systematic reviews have provided prevalence estimates for psychosis,^3^, ^4^ major depression,^3^, ^4^ and substance use disorders among prison populations^3^, ^5^ worldwide and in low-income and middle-income countries,^3^ which are considerably higher than those reported for community-based samples of similar ages.^3^ However, these systematic reviews do not capture the clinical picture of people who are incarcerated and commonly have multiple health problems, particularly co-occurring serious mental illnesses and substance use disorders.^6^, ^7^ Dual disorders (by which we mean a co-occurring serious mental illness and substance use disorder) are key challenges for criminal justice systems^8^ and have been identified as a priority for prison health research.^2^

**Research in context**

**Evidence before this study**

Comorbid mental illnesses and substance use disorders (dual disorders) among people in prison are associated with more severe criminal histories, poorer post-release outcomes, and poorer treatment responses. However, there are uncertainties regarding the burden of dual disorders among prison populations, which is detrimental to service planning. During preparation for this study in February, 2020, we searched Embase, MEDLINE, and PsycINFO without language restrictions for systematic reviews on comorbidity studies in prison settings published between database inception and Feb 28, 2020, using the same search terms as later used in the main systematic review and meta-analysis. We found one systematic review on comorbid post-traumatic stress disorder and mental disorders and one systematic review on comorbid attention-deficit hyperactivity disorder and mental disorders; another systematic review (without meta-analysis) examined the prevalence of comorbid substance use disorders and psychosis or depression in low-income and middle-income countries. The prevalence of dual disorders was not always reported in studies of mental disorders in people in prison. Therefore, we learned that additional efforts would be necessary to identify and collect unpublished data for our analyses.

**Added value of this study**

We have synthesised the current and lifetime prevalence of comorbid serious mental illnesses and substance use disorders among people in prison internationally. We found that the prevalence of current comorbid non-affective psychosis and substance use disorders was 3·5% and the prevalence of current comorbid major depression and substance use disorders was 9·1%. People in prison with substance use disorders had significantly higher odds of comorbid non-affective psychosis or major depression. Around one in two people in prison with non-affective psychosis or major depression had comorbid substance use disorders.

**Implications of all the available evidence**

Imprisonment presents a rare opportunity to reach individuals with dual disorders during a period in their lives in which they have limited access to substances. Prisons typically separate mental health and substance treatment services, and reviewing the effectiveness of these services and how they are linked is required. The degree to which integrated services, screening, and training of staff will improve the early identification and outcomes of dual disorders needs to be researched.

Compared with other people who are incarcerated, those with dual disorders have more serious criminal histories, including previous incarcerations^9^ and violent offences,^10^ and higher rates of serious institutional misconduct while in prison.^10^ Dual disorders are more common among women than among men^9^ and among survivors of childhood physical and sexual abuse^11^ who are involved in the criminal justice system, and they are associated with physical health problems^9^ and impairments in psychosocial, cognitive, and occupational functioning.^12^ After release from prison, individuals with dual disorders have a substantially higher risk of reincarceration,^10^, ^13^ attempting suicide,^14^ and hospitalisation due to injuries than do those without.^15^ Furthermore, having a co-occurring substance use disorder can worsen the prognosis and increase the severity of symptoms of mental illnesses, and is associated with poorer treatment responses and adherence to medication for mental illness.^6^, ^16^, ^17^ Dual disorders pose substantial challenges to treatment planning, and criminal justice systems are often not equipped to address these complexities.^12^

In this systematic review and meta-analysis, we aimed to synthesise the literature on the prevalence of comorbid serious mental illnesses (ie, non-affective psychosis and major depression) and substance use disorders in prison populations worldwide. We aimed to estimate the prevalence of dual disorders among people in prison overall and by alcohol and drug use disorders separately, explore the heterogeneity between estimates, and calculate the association between serious mental illnesses and substance use disorders. The rationale for focusing on these conditions was their high prevalence among people in prison (with one in seven individuals in prison having a serious mental illness),^4^ their common co-occurrence, and their association with poor outcomes in prison and on release, including suicidality and repeat offending.^12^, ^13^, ^14^ In addition, because treating dual disorders requires additional resources and complex interventions, estimating their prevalence will help to guide service development and resource allocation.

## Methods

### Search strategy and selection criteria

This systematic review and meta-analysis followed the Preferred Reporting Items for Systematic Reviews and Meta-Analyses guidelines^18^ for reporting. Comorbid disorders are usually not reported as primary findings in prevalence studies. Therefore, we developed a multi-stage search strategy, first aiming to identify all publications reporting the prevalence of serious mental illnesses and substance use disorders in prison settings. We searched 15 online databases (ASSIA, CAB Abstracts, Criminal Justice Database, Embase, Global Health, Global Index Medicus, IBSS, MEDLINE, NCJRS, PAIS Index, PsycINFO, Russian Science Citation Index, Scielo, Social Services Abstracts, and Web of Science) and the grey literature (Open Grey and ProQuest Dissertations & Theses Global) for literature published between Jan 1, 1980, and Sept 25, 2021. General and database-specific search terms can be found in the appendix (pp 2–5). GB conducted the searches. We considered articles in all languages, and screened the reference lists of all identified papers and relevant systematic reviews.^3^, ^4^, ^5^ Two researchers (SDL and GB) independently screened abstracts and full texts. Where there was disagreement, a third reviewer (APM) was involved in the screening and helped to resolve the disagreement. Articles deemed as relevant but not available in languages understood by the reviewers were translated with Google Translate. We sought summary data. We gathered an initial pool of prevalence studies and screened full texts as to whether they reported the prevalence of dual disorders. If the prevalence of dual disorders was not reported, but the study fulfilled all other inclusion criteria, we contacted the authors by email and asked whether prevalence data could be provided.

Empirical studies meeting the following criteria were included: data were collected from unselected general adult (≥18 years) prison populations applying representative sampling techniques; the prevalence of serious mental illnesses and substance use disorders was reported; disorders were diagnosed in clinical examinations or in interviews by use of validated instruments based on International Classification of Diseases or Diagnostic and Statistical Manual of Mental Disorders criteria; and the prevalence of dual disorders was either reported in the publication or provided after correspondence with study authors. Regarding the diagnosis of substance use disorders, we also included studies that used dependence severity scales with recommended cutoff scores^19^ approximating clinical diagnosis.

Studies were excluded when the target population comprised people who were not in prison at the time of the study or selected groups within prison (eg, minority ethnic individuals or individuals from a particular age group); the sampling strategy was convenient or two-stage (ie, screening followed by a diagnostic interview); mental illnesses and substance use disorders were not assessed or their assessments were based on self-reported instruments without a diagnostic interview; and the prevalence of dual disorders was not reported or provided by the authors. Duplicate publications without relevant additional information, conference abstracts, and studies without empirical data were also excluded.

### Data analysis

Data were manually extracted to Excel sheets according to a predetermined protocol**.** Two reviewers (GB and SDL) independently extracted data from the eligible studies for the following variables: date and country of data collection; sampling method; non-response rate (proportion of potentially eligible individuals who did not participate or did not complete the surveys); type of recruitment (at admission [ie, recruited on arrival to prison] or cross-sectional [ie, recruited across the entire population at variable times during imprisonment]); diagnostic instrument and classification system (International Classification of Diseases; Diagnostic and Statistical Manual of Mental Disorders); interviewer (mental health professional or trained interviewer); sample size; sex; mean age; proportion of participants with previous imprisonment; and number of individuals in prison with dual disorders and each of the single serious mental health disorders and substance use disorders. Furthermore, we extracted data on race and ethnicity, national identity, and country of birth to provide more detailed information on the diverse social groups captured in the samples.

Both current (up to 1 year) and lifetime prevalence were extracted, if available. Our primary outcomes were comorbid non-affective psychosis (International Classification of Diseases F20–29) with substance use disorders (alcohol use disorders [F10] and drug use disorders [F11–19, excluding F17]), and comorbid major depression (F32–33) with substance use disorders (alcohol use disorders and drug use disorders). If prevalence data on non-affective psychosis or major depression were not available, we extracted estimates based on all types of psychoses (F20–29, F31, F32.3, and F33.3) and on affective disorders (F30–39). As a post-hoc secondary outcome, we considered co-occurring axis I disorders (F20–59) and substance use disorders.

Samples capturing both sexes, but not reporting data separately, were either assigned to the majority sex (≥90% of total) or considered as mixed (<90% of total), as per previous work.^3^ We created a dummy variable for samples with small (n≤200) and large (n>200) sample sizes. To account for differences in poorly resourced prison environments and in the access to treatment resources,^3^ we categorised the countries of data collection into high-income and low-income or middle-income countries on the basis of gross national income per capita at the time of data collection^3^ using World Bank Atlas methodology.

The quality of eligible studies was independently appraised by two reviewers (GB and SDL). We applied a questionnaire that has been previously used for the assessment of bias in prison prevalence studies,^3^ with original items largely based on the Joanna Briggs Institute Critical Appraisal Tool for prevalence studies.^20^ Using the modified tool, we assessed the external and internal validity of included studies across ten questions with prespecified response options (appendix p 6). Final scores range between 0 and 10.

The prevalence of dual disorders was pooled with a random-effects meta-analysis, assuming heterogeneity between studies. To overcome challenges posed by proportions being close to, or at the margins of, 0 and 1, we calculated individual sample estimates with score 95% CIs and stabilised variances with the Freeman–Tukey double arcsine transformation to approximate normal distribution. Random-effects models were fitted via the restricted maximum likelihood method to estimate heterogeneity variance,^21^ and the 95% CIs of summary measures were calculated with the Knapp–Hartung variance estimator. Inconsistency was quantified with *I*^2^ to describe the percentage of variation attributed to between-sample heterogeneity, with values higher than 75% indicating considerable heterogeneity.^22^ Assuming considerable between-sample heterogeneity,^3^ we provide ranges of estimates. Current prevalence and lifetime prevalence of dual disorders were separately pooled. If prevalence estimates of dual disorders based on non-affective psychosis and major depression were unavailable, we included estimates in the main analyses derived from all types of psychosis and affective disorders (broad disorder criteria). However, in a post-hoc sensitivity analysis, we excluded estimates for disorders that did not match the primary outcomes exactly (narrow disorder criteria).

Heterogeneity between estimates was assessed by use of random-effects meta-regression across prespecified sample characteristics (ie, sex, sample size, mean age, year of data collection, non-response rate, type of recruitment, country income level, diagnostic classification, and previous imprisonment) and data source (published or unpublished). We did univariate meta-regression when at least ten samples were available,^22^ and adjusted p values for false discovery rate to reduce type I error. If two or more explanatory variables remained significant or close to the significance level (p_adj_≤0·05) after adjustment for multiple comparisons and there were at least 20 estimates available, variables were retained for multivariate meta-regression.

To explore the association between serious mental illnesses and substance use disorders, we computed odds ratios (ORs) and their 95% CIs from the number of individuals in prison with comorbid and single disorders. ORs higher than 1 indicate that dual disorders are more likely to occur than single disorders. Random-effects models were fitted with the restricted maximum likelihood variance estimator and Knapp–Hartung corrections. To avoid computational errors when one or more cells in the 2 × 2 tables included the value of 0, we applied continuity correction of 0·5 in studies with cell frequencies of 0.^22^

We assessed bias in three ways. First, funnel plots were drawn, visualising transformed proportions against their SEs, and the degree of funnel plot asymmetry was evaluated with Egger's test^22^ when at least ten estimates were available. Second, we tested whether samples with lower quality scores systematically distorted pooled estimates by entering the quality score into the meta-regression as a covariate.^3^ Finally, the effect of removing outlier estimates, for which the 95% CI did not overlap with the 95% CI of the pooled effect, from the dataset was analysed. All analyses were done in R (version 4.1.0) by use of the *meta* and *dmetar* packages. The study protocol was registered in PROSPERO, CRD42020207301.

### Role of the funding source

The funders of the study had no role in study design, data collection, data analysis, data interpretation, or writing of the report.

## Results

**Figure 1:**
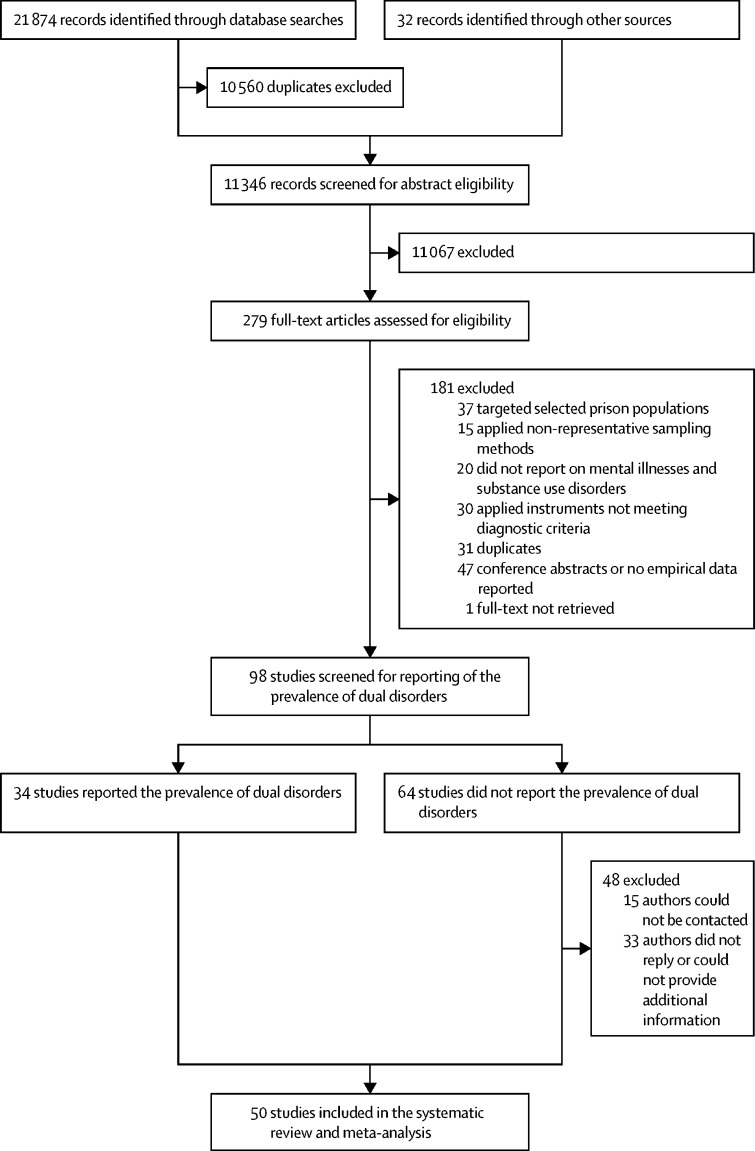
Study selection

Of 11 346 records screened, we identified 34 studies reporting the prevalence of dual disorders among individuals involved in the criminal justice system (figure 1). We corresponded with the authors of 47 publications and received unpublished data on the prevalence of dual disorders for 14 additional studies (33 authors did not answer or could not provide data); furthermore, raw data for two studies are held by review authors and were reanalysed, resulting in unpublished data from a total of 16 studies (figure 1). Overall, we included 50 studies with 59 samples reporting on a total of 24 915 individuals living in prison from 21 countries (Australia,^23^, ^24^, ^25^, ^26^ Brazil,^27^ Burkina Faso,^28^ Canada,^29^, ^30^, ^31^, ^32^, ^33^, ^34^ Chile,^35^, ^36^ China,^37^ Ecuador,^38^ France^39^, ^40^, ^41^ [and French Guiana],^42^ Germany,^43^, ^44^, ^45^, ^46^ Greece,^47^ India,^48^, ^49^ Iran,^50^ Ireland,^51^, ^52^, ^53^, ^54^ Italy,^55^, ^56^ Malaysia,^57^ New Zealand,^58^, ^59^ South Africa,^60^ Spain,^61^, ^62^, ^63^ Uganda,^64^ the UK,^19^ and the USA^65^, ^66^, ^67^, ^68^, ^69^, ^70^, ^71^). Of the pooled total study population, 7612 (30·6%) of 24 915 individuals were women and 17 303 (69·4%) were men, the dual disorder data for 7509 (30·1%) individuals had not previously been published, and 11 studies (n=7010) were from low-income and middle-income countries (appendix pp 7–8). Of 50 included studies, 25 (50%) reported a sample distribution for race and ethnicity, 11 (22%) did not provide any information on social groups, ten (20%) reported on country of birth, and seven (14%) reported on nationality (percentages do not add up to 100% due to multiple reporting; appendix pp 9–10). The quality score of included studies ranged between 4 and 10, with a mean score of 7·8 (SD 1·2; appendix p 11).

**Figure 2:**
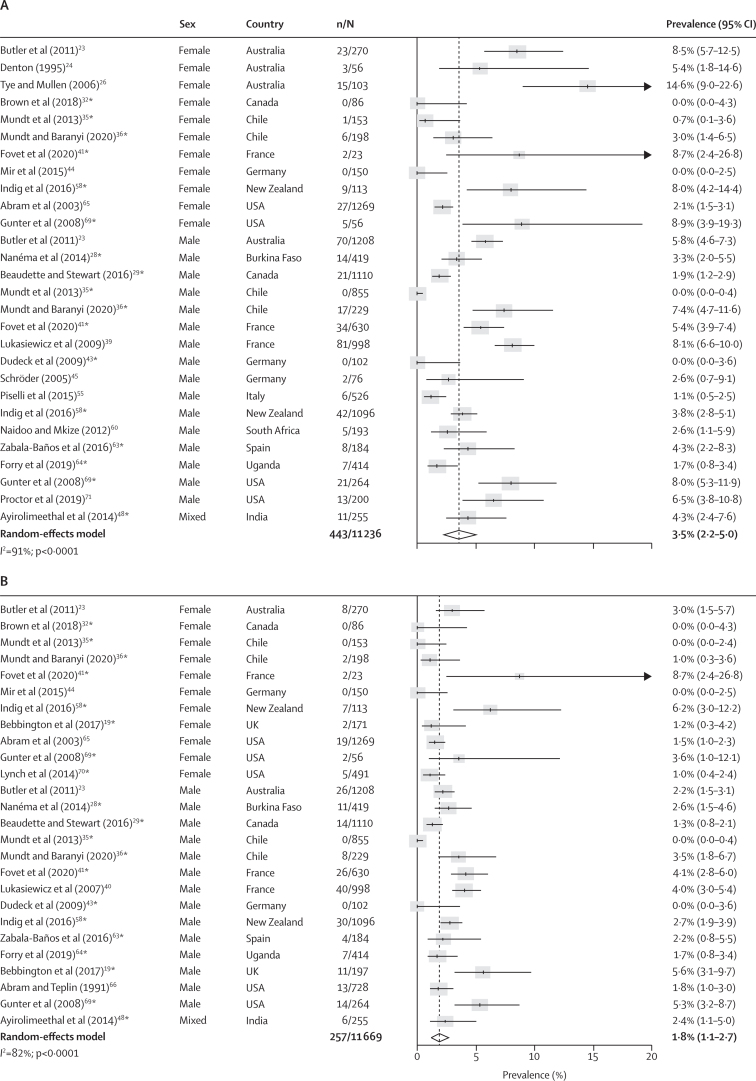

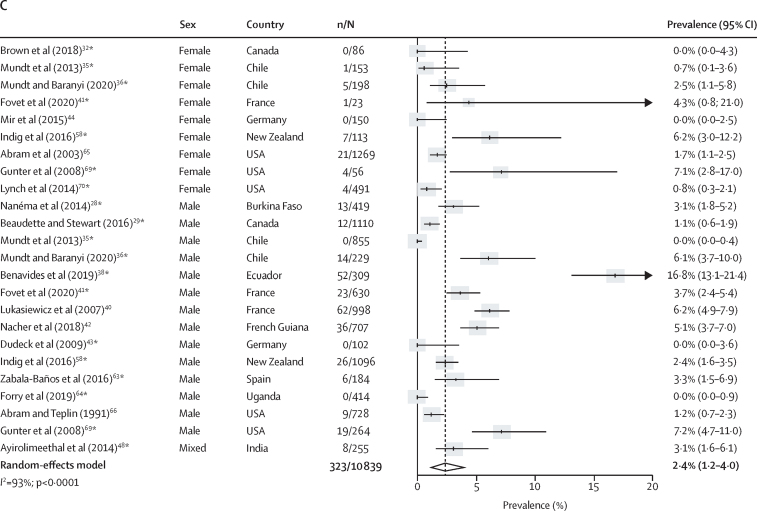
Current prevalence of comorbid non-affective psychosis and substance use disorders in prison populations (A) Substance use disorders. (B) Alcohol use disorders. (C) Drug use disorders. Individual estimates are sorted by sex and country, and were pooled with random-effects meta-analysis. *Estimates are based on previously unpublished data.

Co-occurring non-affective psychosis and any type of substance use disorder was reported in 43 studies.^19^, ^23^, ^24^, ^25^, ^26^, ^28^, ^29^, ^30^, ^32^, ^33^, ^34^, ^35^, ^36^, ^37^, ^38^, ^39^, ^40^, ^41^, ^42^, ^43^, ^44^, ^45^, ^46^, ^48^, ^50^, ^51^, ^52^, ^53^, ^54^, ^55^, ^58^, ^59^, ^60^, ^62^, ^63^, ^64^, ^65^, ^66^, ^67^, ^68^, ^69^, ^70^, ^71^ The pooled current prevalence of co-occurring non-affective psychosis and substance use disorders was 3·5% (95% CI 2·2–5·0; n=11 236; number of included estimates [k]=28), which represented 443 (49·2%) of 900 people with non-affective psychosis and 443 (9·2%) of 4793 people with substance use disorders (figure 2; appendix p 12). Co-occurring current non-affective psychosis and alcohol use disorders was present in 1·8% (95% CI 1·1–2·7; n=11 669; k=26) of the prison population and co-occurring current non-affective psychosis with drug use disorders was present in 2·4% (1·2–4·0; n=10 839; k=24) of the prison population (figure 2). Lifetime prevalences of non-affective psychosis co-occurring with substance, alcohol, or drug use disorders were 6·9% (95% CI 4·7–9·4), 4·9% (2·4–8·0), and 5·3% (2·3–9·4), respectively, affecting the majority of the prison population with a lifetime diagnosis of non-affective psychosis (appendix p 12). Significantly higher rates of current and lifetime non-affective psychosis were found among people in the criminal justice system with substance, alcohol, or drug use disorders compared with individuals living in prison without these conditions (OR 1·7, 95% CI 1·4–2·2 for comorbid current non-affective psychosis and substance use disorders; appendix p 12). Between-sample heterogeneity was high for estimates of current and lifetime prevalence of comorbid non-affective psychosis and substance use disorders (*I*^2^ >80%), which was not explained by any tested factor in our meta-regression after adjustment for multiple comparisons (appendix p 13).

**Figure 3:**
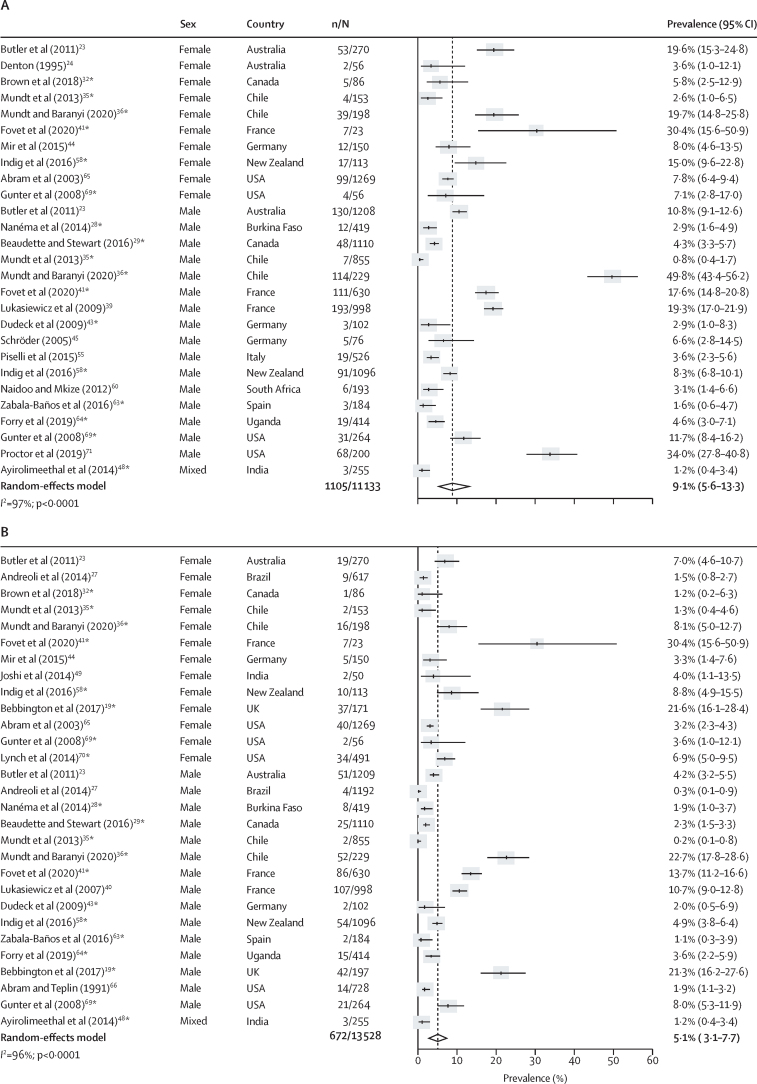

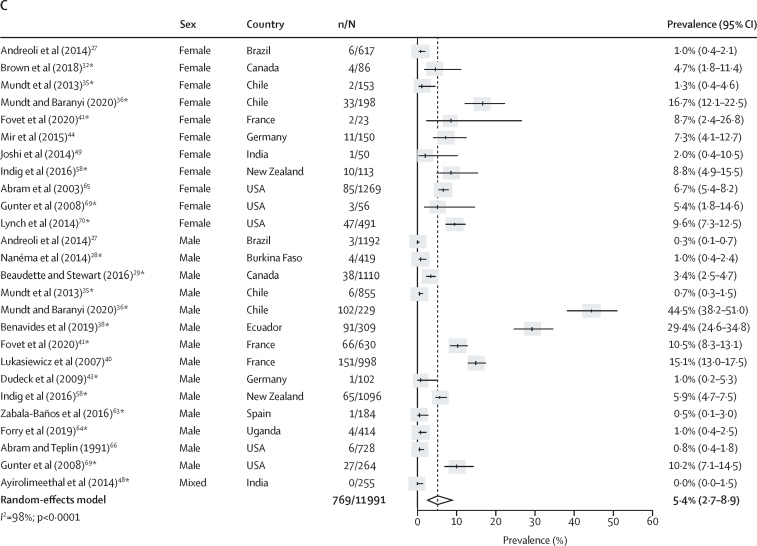
Current prevalence of comorbid major depression and substance use disorders in prison populations (A) Substance use disorders. (B) Alcohol use disorders. (C) Drug use disorders. Individual estimates are sorted by sex and country, and were pooled with random-effects meta-analysis. *Estimates are based on previously unpublished data.

Co-occurring major depression and any type of substance use disorder was reported in 42 studies.^19^, ^23^, ^24^, ^25^, ^27^, ^28^, ^29^, ^30^, ^31^, ^32^, ^33^, ^34^, ^35^, ^36^, ^37^, ^38^, ^39^, ^40^, ^41^, ^43^, ^44^, ^45^, ^46^, ^48^, ^49^, ^50^, ^51^, ^55^, ^57^, ^58^, ^59^, ^60^, ^62^, ^63^, ^64^, ^65^, ^66^, ^67^, ^68^, ^69^, ^70^, ^71^ The prevalence of current co-occurring major depression and substance use disorders was 9·1% (95% CI 5·6–13·3; n=11 133; k=27), representing 1105 (51·6%) of 2143 people with major depression and 1105 (23·3%) of 4733 with substance use disorders (figure 3; appendix p 12). The pooled prevalence of current major depression co-occurring with alcohol use disorders was 5·1% (95% CI 3·1–7·7; n=13 528; k=29) and the pooled prevalence of current major depression co-occurring with drug use disorders was 5·4% (2·7–8·9; n=11 991; k=26; figure 3). Lifetime prevalences of major depression co-occurring with substance, alcohol, or drug use disorders were 22·2% (95% CI 16·9–28·0), 12·4% (7·9–17·8), and 14·3% (9·5–19·8), respectively (appendix p 12). Significantly higher rates of current and lifetime major depression were found among people in the criminal justice system with substance, alcohol, or drug use disorders compared with individuals in prison without these conditions (OR 1·6, 95% CI 1·3–2·0 for comorbid major depression and substance use disorders; appendix p 12). Between-sample heterogeneity was very high (*I*^2^ >90%) for estimates of current and lifetime comorbid major depression and substance use disorders; we found that a higher non-response rate was associated with higher lifetime comorbidity between major depression and alcohol use disorders in univariate meta-regression (b [unstandardised coefficient]=0·007; SE=0·002; p_adj_=0·040; appendix p 14).

We included 24 studies reporting comorbidity between axis I disorders and substance use disorders.^23^, ^24^, ^26^, ^28^, ^29^, ^32^, ^33^, ^34^, ^35^, ^36^, ^38^, ^39^, ^41^, ^43^, ^47^, ^51^, ^56^, ^58^, ^61^, ^62^, ^65^, ^66^, ^69^, ^70^ The current prevalence of co-occurring axis I disorders and substance use disorders was 20·7% (95% CI 13·8–28·5; n=10 998; k=24), representing 2205 (51·4%) of 4293 people with axis I disorders and 2205 (47·9%) of 4600 people with substance use disorders (appendix p 12). The current prevalence of comorbid axis I disorders and alcohol use disorders was 11·1% (95% CI 6·6–16·5; n=8809; k=18) and the current prevalence of comorbid axis I disorders and drug use disorders was 15·8% (8·9–24·2; n=7640; k=17; appendix p 12). The lifetime prevalence of comorbid axis I disorders and substance use disorders was 39·9% (95% CI 28·3–52·1), the lifetime prevalence of comorbid axis I disorders and alcohol use disorders was 27·9% (16·0–41·7), and the lifetime prevalence of comorbid axis I disorders and drug use disorders was 29·7% (14·3–47·9; appendix p 12). Current and lifetime axis I disorders were significantly associated with substance, alcohol, and drug use disorders (appendix p 12). Between-sample heterogeneity was very high for current and lifetime estimates of comorbid axis I disorders and substance use disorders (*I*^2^>90%; appendix p 12). Multivariate meta-regression indicated a higher current prevalence of comorbid axis I disorders and substance use disorders with recruitment at admission to prison compared with cross-sectional samples, and among investigations with higher non-response rates (appendix p 15). Due to the low number of estimates, multivariate meta-regression could not be done for the lifetime prevalence of comorbid axis I disorders and substance use disorders; univariate meta-regression suggested that the lifetime prevalence of comorbid axis I disorders and substance use disorders increased with the recency of data collection and was higher in investigations with higher non-response rates (appendix p 15). Meta-regression did not indicate any differences in the prevalences of dual disorders between high-income and low-income and middle-income countries (appendix pp 13–15).

Excluding estimates that were based on broad diagnostic criteria for psychosis (ie, affective and non-affective) and affective disorders in a post-hoc sensitivity analysis did not materially change the findings (appendix p 16). Egger's test only found evidence of funnel plot asymmetry for the lifetime prevalence of co-occurring major depression and alcohol use disorders (bias 6·445 [SE 2·348]; p_bias_=0·017; appendix p 17). After visually inspecting the respective funnel plots (appendix pp 18–20) and excluding one large sample with a very small prevalence estimate,^37^ the overall prevalence of lifetime co-occuring major depression and alcohol use disorders increased (13·5%, 95% CI 9·0–18·8) and Egger's test did not indicate publication bias (p_bias_=0·15). Adding the quality score as a covariate to the meta-regression did not signal systematic bias towards lower quality studies (appendix p 21). Excluding outlier estimates, for which the 95% CI did not overlap with the 95% CI of the pooled prevalence, from the meta-analysis led to a large decrease in heterogeneity (appendix p 22). Still, prevalence estimates remained close to those reported in the main findings, without any clear pattern of increases or decreases (appendix p 22).

## Discussion

This systematic review and meta-analysis reported on the prevalence of co-occurring serious mental illnesses and substance use disorders among prison populations worldwide. Analyses were based on 24 915 individuals and included unpublished comorbidity data from 16 studies. Our findings show the high burden of dual disorders among individuals involved in the criminal justice system: comorbid substance use disorders with non-affective psychosis, major depression, or axis I disorders were currently present in 3·5%, 9·1%, and 20·7% of the prison population, respectively. We found evidence for the higher prevalence of dual disorders in studies with higher non-response rates (lifetime prevalence of comorbid major depression and alcohol use disorders), with recruitment at prison intake (current prevalence of comorbid axis I disorders and substance use disorders), and with more recent data collections (lifetime prevalence of comorbid axis I disorders and substance use disorders).

The current prevalence of comorbid non-affective psychosis and substance use disorders was approximately 20-times higher in prison populations in our analysis than in general populations from other studies;^6^, ^72^ comorbid major depression and substance use disorders was about twice as prevalent in prison samples in our analysis than in community samples from a previously published analysis.^73^ In the general population, substance use disorders are common among individuals with schizophrenia spectrum disorders^6^ and major depression;^7^ prevalences are generally higher for illicit drug use than for alcohol use disorders. There is some evidence suggesting that mental disorders typically start at an earlier age than substance use disorders.^17^ As the disorders interact, it can be challenging to establish causal pathways. Potential interactions include: (1) mental disorders lead to substance use disorders (eg, self-medication to alleviate symptoms); (2) substance use contributes to the onset or persistence of mental disorders (eg, substance-induced conditions); or (3) mental disorders and substance use disorders share common genetic vulnerabilities or risk factors, or involve similar brain regions.^6^, ^7^, ^8^, ^17^, ^74^

We found evidence for the higher prevalence of dual disorders in studies with higher non-response rates, which has also been reported in a previous systematic review on the prevalence of substance use disorders in prison populations.^5^ We found a higher prevalence of current dual disorders in studies recruiting at admission to prison compared with cross-sectional investigations, which has also been shown previously for single disorders^3^ and probably reflects the reduced availability of psychoactive substances in prisons. Our finding of a higher lifetime prevalence of comorbid axis I disorders and substance use disorders in more recent investigations might be explained by the trend in the past decades of an increasing prevalence of drug use disorders^5^ and depression^4^ in prisons. Although we report meta-regression findings after adjusting for false discovery rates, the same explanatory variables seemed to be associated with the prevalence of dual disorders before applying corrections, indicating that these findings probably cannot be explained by chance.

Several limitations of this systematic review and meta-analysis should be considered. Despite making efforts to contact all authors of relevant primary studies, some authors of older studies could not be contacted (eg, if the corresponding email address was not provided) and raw data were sometimes no longer available. Furthermore, the heterogeneity between samples was high, which is not uncommon in meta-analyses of prevalence data.^3^, ^4^ We did a range of sensitivity analyses, which were consistent with the main findings and explained heterogeneity to a limited extent.

The findings of this systematic review and meta-analysis have several implications. About half of the prison population with serious mental illnesses had comorbid substance use disorders. Dual disorders are associated with poorer treatment outcomes compared with single disorders^17^ and the detection rates of comorbid conditions in the criminal justice system are low.^12^ Comprehensive assessment of dual disorders is required when individuals enter the criminal justice system, including recording the sequence of onset of mental health disorders and substance use disorders and screening for psychosocial problems and traumatic experiences (eg, violence and abuse).^12^, ^75^ Early identification of dual disorders can help to identify the specialised care needed without further delay, avoid inappropriate treatment planning, and increase overall treatment success.^12^ As screening for dual disorders is not often routinely done in prison, specific policies should encourage screening and the training of health-care staff who work in the criminal justice system in dual disorders should be strengthened.^12^, ^75^ Integrated approaches are needed instead of parallel or sequential treatments for mental health and substance use disorders.^75^ Even in prisons located in high-income countries, comprehensive treatment options are sparse.^12^, ^75^ A US investigation found that 7076 (38·4%) of 18 421 individuals living in prison received any behavioural treatment and only 1299 (7·1%) received treatment for substance use and mental health problems;^76^ in low-income and middle-income countries, the unmet need is probably higher due to resource constraints. Developing and establishing cost-effective and scalable interventions could have a considerable impact on treatment provision in prison settings.^3^ Individuals in prison with mental and substance use disorders have an increased risk of suicide compared with individuals in prison without these disorders,^77^ and there is some evidence suggesting that those with dual disorders have the highest risk.^14^ Future research should further examine the link between dual disorders and suicide risk in prison settings. Reporting minority status in future research and assessing dual disorders in these groups^65^ requires further attention.

Co-occurring disorders pose considerable challenges for adequate screening, identification, treatment planning, and long-term management. This systematic review and meta-analysis indicates the high burden of dual disorders among people who are incarcerated. Meeting the complex unmet treatment needs of prison populations with dual disorders is not only a challenge for criminal justice, but for the mental health and public health services more widely. Furthermore, imprisonment is an opportunity for the identification and treatment of individuals who are difficult to reach in the community during a time window when they have limited access to substances.

## Data sharing

The extracted data that support the findings of this study are available upon reasonable request of researchers to the corresponding author (APM; adrian.mundt@mail.udp.cl). The time of data availability is undefined and corresponds to good clinical practice. The study protocol has been published on PROSPERO, https://www.crd.york.ac.uk/prospero/display_record.php?RecordID=207301.

## Declaration of interests

SF provided expert clinical reports on suicide risk and deaths in custody and is an expert member of the UK's Independent Advisory Panel on Deaths in Custody. All other authors declare no competing interests.
