# Supplementary material for: The prevalence of comorbid serious mental illnesses and substance use disorders in prison populations: a systematic review and meta-analysis
Source: Lancet Public Health. 2022 Jun 1;7(6):e557–68. doi: 10.1016/S2468-2667(22)00093-7 (PMC9178214; doi:10.1016/S2468-2667(22)00093-7)
Supplement: Supplementary appendix [file mmc1.pdf]

THE LANCET
Public Health

Supplementary appendix

This appendix formed part of the original submission and has been peer reviewed. We post it as supplied by the authors.

Supplement to: Baranyi G, Fazel S, Delhey Langerfeldt S, Mundt AP. The prevalence of comorbid serious mental illnesses and substance use disorders in prison populations: a systematic review and meta-analysis. *Lancet Public Health* 2022; **7:** 557–68.

Web Appendix

	Page
Supplementary Material 1: General search terms …………………………………………………..	2
Supplementary Table 1: Search terms executed in databases ………………………………………….	3-5
Supplementary Material 2: Critical Appraisal Tool ………………………………………………...	6
Supplementary Table 2: Characteristics of included samples ..…………………………….………	7-8
Supplementary Table 3: Race/ethnicity, nationality and country of birth in the included studies ……..	9-10
Supplementary Table 4: Quality appraisal of included samples ……………………………………...	11
Supplementary Table 5: Prevalence estimates and odds ratios of co-occurring mental and substance use disorders in prison populations ………………………………………………………………..	12
Supplementary Table 6: Meta-regression exploring heterogeneity across estimates of comorbid non-affective psychosis and substance use disorders ……………………………………………………	13
Supplementary Table 7: Meta-regression exploring heterogeneity across estimates of comorbid depression and substance use disorders ……………………………………………………………..…	14
Supplementary Table 8: Meta-regression exploring heterogeneity across estimates of comorbid Axis I and substance use disorders ………………………………………………………………….	15
Supplementary Table 9: Prevalence estimates and odds ratios of comorbid mental and substance use disorders applying narrow disorder criteria for non-affective psychosis and major depression ……….	16
Supplementary Table 10: Egger’s test indicating funnel plot asymmetry ……………………………	17
Supplementary Figure 1: Funnel plots for co-occuring substance use disorders and (1) non-affective psychosis, (2) depression and (3) Axis I disorders ………………………………………………….	18-20
Supplementary Table 11: Meta-regression with quality appraisal score ………………………………	21
Supplementary Table 12: Prevalence of comorbid mental and substance use disorders before and after exclusion of outlier estimates ……………………………………………………………….	22
References …………………………………………………………………………………….	23-26

Supplementary Material 1: General search terms

Mental disorders

Mental Disorders (Subject Heading) OR Substance-Related Disorders (Subject Heading) OR psychiatr* OR psycho* OR depress* OR schizophren* OR alcohol* OR substance OR addict* OR "dual disorder" OR "dual disorders"

Prison setting

prison* OR inmate* OR imprison* OR correctional* OR jail* OR "criminal justice" OR detain* OR sentenced OR remand OR incarcerate*

Epidemiological studies

Epidemiology (Subject Heading) OR comorbid* OR prevalence OR incidence

Limit: 1980 to Present

Supplementary Table 1: Search terms executed in databases

Prison	Epidemiology	Mental health
**Applied Social Sciences Index and Abstracts (ASSIA) (25/09/2021) (1006)***		
ab,ti,su(prison* or inmate* or imprison* or correctional* or jail* or "criminal justice" or detain* or sentenced or remand or incarcerate*) 30530	Exact("epidemiology") OR ab,ti,su(comorbid* OR prevalence OR incidence) 78171	Exact("substance-related disorders" OR "substance abuse disorders" OR "mental disorders") or ab,ti,su(psychiatr* or psycho* or depress* or schizophren* or alcohol* or substance or addict* or "dual disorder" or "dual disorders") 293200
**CAB Abstracts (25/09/2021) (185)***		
(prison* or inmate* or imprison* or correctional* or jail* or "criminal justice" or detain* or sentenced or remand or incarcerate*)·mp· [mp=abstract, title, original title, broad terms, heading words, identifiers, cabicodes] 2969	epidemiology/ or comorbid*·mp· or prevalence·mp· or incidence·mp· [mp=abstract, title, original title, broad terms, heading words, identifiers, cabicodes] 559202	mental disorders/ or substance abuse/ or psychiatr*·mp· or psycho*·mp· or depress*·mp· or schizophren*·mp· or alcohol*·mp· or substance·mp· or addict*·mp· or "dual disorder"·mp· or "dual disorders"·mp· [mp=abstract, title, original title, broad terms, heading words, identifiers, cabicodes] 358832
**Criminal Justice Database (25/09/2021) (894)***		
ab,ti,su(prison* or inmate* or imprison* or correctional* or jail* or "criminal justice" or detain* or sentenced or remand or incarcerate*) 160615	Exact("epidemiology") OR ab,ti,su(comorbid* OR prevalence OR incidence) 21219	Exact("substance-related disorders" OR "substance abuse" OR "mental disorders") OR ab,ti,su(psychiatr* OR psycho* OR depress* OR schizophren* OR alcohol* OR substance OR addict* OR "dual disorder" OR "dual disorders") 123384
**Embase (25/09/2021) (4797)***		
(prison* or inmate* or imprison* or correctional* or jail* or "criminal justice" or detain* or sentenced or remand or incarcerate*)·mp· [mp=title, abstract, heading word, drug trade name, original title, device manufacturer, drug manufacturer, device trade name, keyword heading word, floating subheading word, candidate term word] 52798	epidemiology/ or comorbid*·mp· or prevalence·mp· or incidence·mp· [mp=title, abstract, heading word, drug trade name, original title, device manufacturer, drug manufacturer, device trade name, keyword heading word, floating subheading word, candidate term word] 2802900	mental disease/ or substance abuse/ or drug dependence/ or drug abuse/ or alcohol abuse/ or psychiatr*·mp· or psycho*·mp· or depress*·mp· or schizophren*·mp· or alcohol*·mp· or substance·mp· or addict*·mp· or "dual disorder"·mp· or "dual disorders"·mp· [mp=title, abstract, heading word, drug trade name, original title, device manufacturer, drug manufacturer, device trade name, keyword heading word, floating subheading word, candidate term word] 3308760
**Global Health (25/09/2021) (813)***		
(prison* or inmate* or imprison* or correctional* or jail* or "criminal justice" or detain* or sentenced or remand or incarcerate*).mp. [mp=abstract, title, original title, broad terms, heading words, identifiers, cabicodes] 7057	epidemiology/ or comorbid*.mp. or prevalence.mp. or incidence.mp. [mp=abstract, title, original title, broad terms, heading words, identifiers, cabicodes] 637009	mental disorders/ or substance abuse/ or psychiatr*.mp. or psycho*·mp· or depress*·mp· or schizophren*·mp· or alcohol*·mp· or substance·mp. or addict*.mp. or "dual disorder".mp. or "dual disorders".mp. [mp=abstract, title, original title, broad terms, heading words, identifiers, cabicodes] 300699
**International Bibliography of the Social Sciences (IBSS) (25/09/2021) (296)***		
ab,ti,su(prison* or inmate* or imprison* or correctional* or jail* or "criminal justice" or detain* or sentenced or remand or incarcerate*) 50713	Exact("epidemiology") OR ab,ti,su(comorbid* OR prevalence OR incidence) 41675	Exact("substance-related disorders" OR "substance abuse" OR "mental disorders") or ab,ti,su(psychiatr* or psycho* or depress* or schizophren* or alcohol* or substance or addict* or "dual disorder" or "dual disorders") 206476
**Global Index Medicus (GIM) (25/09/2021) (277)***		
tw:(prison* or inmate* or imprison* or correctional* or jail* or "criminal justice" or detain* or sentenced or remand or incarcerate*) 3825	tw:(epidemiolog* OR comorbid* OR prevalence OR incidence) 320363	tw:("mental illness" or "mental disorder" OR "mental disorders" OR "drug use disorder" OR psychiatr* or psycho* or depress* or schizophren* or alcohol* or substance or addict* or "dual disorder" or "dual disorders") 209699
**Ovid MEDLINE(R) and Epub Ahead of Print, In-Process, In-Data-Review & Other Non-Indexed Citations, Daily and Versions(R) (25/09/2021) (3727)***		
(prison* or inmate* or imprison* or correctional* or jail* or "criminal justice" or detain* or sentenced or remand or incarcerate*).mp. [mp=title, abstract, original title, name of substance word, subject heading word, floating	Epidemiology/ or comorbid*.mp. or prevalence.mp. or incidence.mp. [mp=title, abstract, original title, name of substance word, subject heading word, floating sub-heading word, keyword heading word, organism supplementary	Mental Disorders/ or Substance-Related Disorders/ or psychiatr*.mp. or psycho*.mp. or depress*.mp. or schizophren*.mp. or alcohol*.mp. or substance.mp. or addict*.mp. or "dual disorder".mp. or "dual disorders".mp. [mp=title, abstract, original

sub-heading word, keyword heading word, organism supplementary concept word, protocol supplementary concept word, rare disease supplementary concept word, unique identifier, synonyms] 44424	concept word, protocol supplementary concept word, rare disease supplementary concept word, unique identifier, synonyms] 1805551	title, name of substance word, subject heading word, floating sub-heading word, keyword heading word, organism supplementary concept word, protocol supplementary concept word, rare disease supplementary concept word, unique identifier, synonyms] 3017644
**National Criminal Justice Reference Service (NCJRS) Abstracts Database (25/09/2021) (597)***		
ab,ti,su(prison* or inmate* or imprison* or correctional* or jail* or "criminal justice" or detain* or sentenced or remand or incarcerate*) 74727	ab,ti,su(comorbid* OR prevalence OR incidence OR epidemiology) 9818	Exact("substance abuse (60399)" OR "mental disorders (04529)") OR ab,ti,su(psychiatr* OR psycho* OR depress* OR schizophren* OR alcohol* OR substance OR addict* OR "dual disorder" OR "dual disorders") 34647
**PAIS Index (25/09/2021) (231)***		
ab,ti,su(prison* or inmate* or imprison* or correctional* or jail* or "criminal justice" or detain* or sentenced or remand or incarcerate*) 21432	Exact("epidemiology") or ab,ti,su(comorbid* OR prevalence OR incidence) 22449	Exact("substance-related disorders" OR "mental disorders") or ab,ti,su(psychiatr* or psycho* or depress* or schizophren* or alcohol* or substance or addict* or "dual disorder" or "dual disorders") 40755
**APA PsycInfo (25/09/2021) (3536)***		
(prison* or inmate* or imprison* or correctional* or jail* or "criminal justice" or detain* or sentenced or remand or incarcerate*).mp. [mp=title, abstract, heading word, table of contents, key concepts, original title, tests & measures, mesh] 57499	exp Epidemiology/ or comorbid*.mp. or prevalence.mp. or incidence.mp. [mp=title, abstract, heading word, table of contents, key concepts, original title, tests & measures, mesh] 272908	exp "Substance Use Disorder"/ or exp Mental Disorders/ or psychiatr*.mp. or psycho*.mp. or depress*.mp. or schizophren*.mp. or alcohol*.mp. or substance.mp. or addict*.mp. or "dual disorder".mp. or "dual disorders".mp. [mp=title, abstract, heading word, table of contents, key concepts, original title, tests & measures, mesh] 2235079
**Russian Science Citation Index (25/09/2021) (37)***		
TS=(prison* or inmate* or imprison* or correctional* or jail* or "criminal justice" or detain* or sentenced or remand or incarcerate*) 2980	TS=(epidemiolog* OR comorbid* OR prevalence OR incidence) 29745	TS=("mental illness" or "mental disorder" OR "mental disorders" OR "drug use disorder" OR psychiatr* or psycho* or depress* or schizophren* or alcohol* or substance or addict* or "dual disorder" or "dual disorders") 50269
**Scielo (25/09/2021) (137)***		
TS=(prison* or inmate* or imprison* or correctional* or jail* or "criminal justice" or detain* or sentenced or remand or incarcerate*) 4382	TS=(epidemiolog* OR comorbid* OR prevalence OR incidence) 65513	TS=("mental illness" or "mental disorder" OR "mental disorders" OR "drug use disorder" OR psychiatr* or psycho* or depress* or schizophren* or alcohol* or substance or addict* or "dual disorder" or "dual disorders") 59441
**Social Services Abstracts (25/09/2021) (343)***		
ab,ti,su(prison* or inmate* or imprison* or correctional* or jail* or "criminal justice" or detain* or sentenced or remand or incarcerate*) 11736	Exact("epidemiology") OR ab,ti,su(comorbid* OR prevalence OR incidence) 20114	Exact("mental illness" OR "substance-related disorders" OR "substance abuse disorders" OR "mental disorders") or ab,ti,su(psychiatr* or psycho* or depress* or schizophren* or alcohol* or substance or addict* or "dual disorder" or "dual disorders") 76816
**Web of Science Core Collection (25/09/2021) (4198)***		
TS=(prison* or inmate* or imprison* or correctional* or jail* or "criminal justice" or detain* or sentenced or remand or incarcerate*) 164807	TS=(epidemiolog* OR comorbid* OR prevalence OR incidence) 2270730	TS=("mental illness" or "mental disorder" OR "mental disorders" OR "drug use disorder" OR psychiatr* or psycho* or depress* or schizophren* or alcohol* or substance or addict* or "dual disorder" or "dual disorders") 3014513
**Grey literature**		
**Open Grey (01/10/2021) (29)†**		
prison* OR inmate* OR imprison* OR correctional* OR jail* OR "criminal justice" OR detain* OR sentenced OR remand OR incarcerate* (2919)	comorbid* OR prevalence OR incidence OR epidemiolog* (14703)	psychiatr* OR psycho* OR depress* OR schizophren* OR alcohol* OR substance OR addict* OR "dual disorder" OR "dual disorders" (71192)
**ProQuest Dissertations & Theses Global* (25/09/2021) (800)***		

ab,ti,su(prison* or inmate* or imprison* or correctional* or jail* or "criminal justice" or detain* or sentenced or remand or incarcerate*) 24,050	Exact("epidemiology") OR ab,ti,su(comorbid* OR prevalence OR incidence) 99,901	Exact("mental disorders") OR "drug use disorder" OR psychiatr* or psycho* or depress* or schizophren* or alcohol* or substance or addict* or "dual disorder" or "dual disorders" 1,953,032

*Number after discharging duplicates.

†Hits were not exported in reference manager.

Supplementary Material 2: Critical Appraisal Tool

Reviewer: ____________________ Date: __________________

Author: ____________________ Year: _______ Record No. _______

No.	Item	Rating
**External Validity**		
1.	Was the study's target population a close representation of the national/regional population? † 1 point: Samples from multiple institutions, or sample is representative for a province or for the whole country. 0 point: Otherwise.	
2.	Were study participants recruited in an appropriate way?* 1 point: Stratified random, random, population or systematic sampling. 0 point: Convenience sampling.	
3.	Was the sample size adequate?* 1 point: Sample size equal to or greater than 200 participants or a pre-calculated sample size reached. 0 point: Otherwise.	
4.	Was the data analysis conducted with sufficient coverage of the identified sample?* 1 point: Participation rate was equal or higher than 75% or non-response analysis indicated that the sample was representative for the target population. 0 point: Otherwise.	
**Internal Validity**		
5.	Were the study subjects and the setting described in detail?* 1 point: Reporting of the descriptive statistics to describe the sample included gender AND at least three other socio-demographic or criminal characteristic. 0 point: Otherwise.	
6.	Was the same standardized interview used for data collection by all subjects? † 1 point: Yes. 0 point: Other mode of data collection were applied.	
7.	Were objective, standard criteria used for the measurement of the condition?* 1 point: Disorders were assessed according to the criteria of DSM or ICD. 0 point: Conditions were measured with standardized screening scale, which did not fulfil disorder criteria.	
8.	Was the condition measured reliably?* 1 point: Psychiatrist(s)/psychologist(s) made the diagnoses. 0 point: Trained interviewer(s) made the diagnoses.	
9.	Was there appropriate statistical reporting?* 1 point: Confidence intervals for prevalence rates or exact case numbers were reported. 0 point: Only prevalence estimates were reported.	
10.	Were subpopulations identified using objective criteria?* 1 point: The study explored only female/only male prisoners OR the results were reported separately for male/female prisoners. 0 point: The study explored both male and female prisoners, but results were not reported separately.	
	**Score:**	

* taken or adapted from JBI Critical Appraisal Checklist for Studies Reporting Prevalence.[1]

† taken or adapted from the Risk of Bias Tool.[2]

Supplementary Table 2: Characteristics of included samples

Study	Country	Sex	Sample size	Type of recruitment	Sampling method	Diagnostic instrument	Diagnostic criteria	Time coverage	QA
Abram et al (2003)[3]	US	Female	1269	A	Stratified random	DIS-III-R	DSM-III-R	Current	6
Abram et al (1991)[4]	US	Male	728	A	Stratified random	NIMH-DIS	DSM-III-R	Lifetime/current	8
Alevizopoulos and Igoumenou (2016)[5]	Greece	Male	495	C	Systematic	ISPI	Not stated	Current	8
Andreoli et al (2014)[6]†	Brazil	Male	1192	C	Stratified random	CIDI	ICD-10	Lifetime/current	7
Andreoli et al (2014)[6]†	Brazil	Female	617	C	Stratified random	CIDI	ICD-10	Lifetime/current	8
Assadi et al (2006)[7]	Iran	Male	351	C	Stratified random	SCID-CV	DSM-IV	Lifetime	9
Ayirolimeethal et al (2014)[8]*	India	Mixed	255	A	Population	MINI-Plus	Not stated	Current	7
Beaudette and Stewart (2016)[9]*	Canada	Male	1110	A	Population	SCID-I	DSM-IV	Lifetime/current	9
Bebbington et al (2017)[10]*	UK	Male	197	C	Sequential random	CIS-R, SCAN, SADQ	ICD-10	Current	8
Bebbington et al (2017)[10]*	UK	Female	171	C	Sequential random	CIS-R, SCAN, SADQ	ICD-10	Current	8
Benavides et al (2019)[11]*	Ecuador	Male	309	C	Random	MINI	DSM-IV	Current	9
Blanchette and Motiuk, (1996)[12]†	Canada	Female	76	C	Population	DIS	DSM-III	Lifetime	6
Brink et al (2001)[13]	Canada	Male	202	A	Random	SCID-I	DSM-IV	Lifetime	9
Brown et al (2018)a[14]*	Canada	Female	86	A	Population	SCID-I/NP	DSM-IV-TR	Lifetime/current	7
Brown et al (2018)b[15]*	Canada	Female	154	C	Systematic sampling	SCID-I/NP	DSM-IV-TR	Lifetime	7
Butler et al (2011)[16]	Australia	Male	1208	A, C	Random	CIDI-A	ICD-10	Current	7
Butler et al (2011)[16]	Australia	Female	270	A, C	Random	CIDI-A	ICD-10	Current	7
Chiles et al (1990)[17]	US	Male	109	A	Not stated	DIS	DSM-III-R	Lifetime	4
Cote and Hodgins (1990)[18]	Canada	Male	495	C	Random	DIS-III-A	DSM-III	Lifetime	8
Curtin et al (2009)[19]†	Ireland	Male	615	A	Population	SADS-L	ICD-10	Lifetime	10
Daniel et al (1988)[20]	US	Female	100	A	Population	DIS-III	DSM-III	Lifetime	8
Denton (1995)[21]	Australia	Female	56	C	Population	SCID-R	DSM-III-R	Lifetime/current	7
Dudeck et al (2009)[22]*	Germany	Male	102	C	Population	SCID-I	DSM-IV	Lifetime/current	7
Duffy et al (2006)[23]	Ireland	Male	438	C	Stratified random	SADS-L, SODQ	ICD-10	Lifetime	9
Forry et al (2019)[24]*	Uganda	Male	414	C	Simple random	MINI	ICD-10	Current	8
Fovet et al (2020)[25]*	France	Male	630	A	Simple random	MINI	ICD-10	Current	8
Fovet et al (2020)[25]*	France	Female	23	A	Simple random	MINI	ICD-10	Current	7
Gunter et al (2008)[26]*	US	Male	264	A	Simple random	MINI-Plus	DSM-IV	Current	7
Gunter et al (2008)[26]*	US	Female	56	A	Simple random	MINI-Plus	DSM-IV	Current	6
Herrman et al (1991)[27]	Australia	Male	158	C	Stratified random	SCID	DSM-III-R	Lifetime	7
Herrman et al (1991)[27]	Australia	Female	31	C	Stratified random	SCID	DSM-III-R	Lifetime	7
Indig et al (2016)[28]*	New Zealand	Male	1096	A	Population	CIDI	DSM-IV	Lifetime/current	9
Indig et al (2016)[28]*	New Zealand	Female	113	A	Population	CIDI	DSM-IV	Lifetime/current	7
Joshi et al (2014)[29]	India	Female	50	C	Population	Clinical judgement	DSM-IV TR	Current	5
Linehan et al (2005)[30]	Ireland	Male	232	C	Stratified random	SADS-L, SODQ	ICD-10	Lifetime	9

Lopez et al (2016)[31]	Spain	Male	472	C	Random	SCID-I	DSM-IV	Lifetime/current	10
Lukasiewicz et al (2009)[32] & Lukasiewicz et al (2007)[33]	France	Male	998	C	Stratified random	MINI-Plus	DSM-IV	Current	8/7
Lynch et al (2014)[34]*	US	Female	491	C	Random	CIDI, SCID-I	DSM-IV	Lifetime/current	8
Mir et al (2015)[35]	Germany	Female	150	A	Population	MINI	DSM-IV	Current	8
Mundt et al (2013)[36]*	Chile	Male	855	C	Stratified random	CIDI	DSM-IV	Current	9
Mundt et al (2013)[36]*	Chile	Female	153	C	Stratified random	CIDI	DSM-IV	Current	8
Mundt and Baranyi (2020)[37]*	Chile	Male	229	A	Random	MINI	DSM-IV	Current	9
Mundt and Baranyi (2020)[37]*	Chile	Female	198	A	Population	MINI	DSM-IV	Current	8
Nacher et al (2018)[38]	French Guiana	Male	707	A	Population	MINI	DSM-V	Current	9
Naidoo and Mkize (2012)[39]	South Africa	Male	193	C	Stratified random	MINI	ICD-10 and DSM-IV	Current	7
Nanéma et al (2014)[40]*	Burkina Faso	Male	419	C	Systematic random	MINI	ICD-10	Current	7
Piselli et al (2015)[41]	Italy	Male	526	C	Population	SCID-I, ASI-X	DSM-IV	Current	9
Piselli et al (2009)[42]	Italy	Male	302	A	Population	SCID-I	DSM-IV	Current	8
Proctor et al (2019)[43]	US	Male	200	A	Random	CAAPE-5	DSM-V	Current	6
Schröder (2005)[44]	Germany	Male	76	C	Random	SCID-I	DSM-IV	Current	6
Simpson et al (1999)[45]	New Zealand	Mixed	1248	C	Population and stratified random	CIDI-A	DSM-IV	Lifetime	8
Tye and Mullen (2006)[46]	Australia	Female	103	C	Population	SMHWB	ICD-10	Current	9
Vicens et al (2011)[47]†	Spain	Male	707	C	Stratified random	SCID	DSM-IV	Lifetime	8
Widmann (2006)[48]	Germany	Female	63	C	Population	SCID-I	DSM-IV	Lifetime	7
Wright et al (2006)[49]	Ireland	Female	94	A	Population	SADS-L, SODQ	ICD-10	Lifetime	8
Wright et al (2006)[49]	Ireland	Female	92	C	Population	SADS-L, SODQ	ICD-10	Lifetime	9
Zabala-Baños et al (2016)[50]*	Spain	Male	184	C	Stratified random	SCID-I	DSM-IV	Lifetime/current	8
Zamzam and Hatta (2000)[51]	Malaysia	Female	80	A, C	Population	CIDI	Not stated	Lifetime	7
Zhong et al (2020)[52]	China	Female	2703	C	Population	MINI	DSM-IV	Lifetime	9

ASI-X=Addiction Severity Index; AUDIT=Alcohol Use Disorders Identification Test; A=admission; CAAPE=Comprehensive Addictions and Psychological Evaluation; C=cross-sectional; CIDI=Composite International Diagnostic Interview; DIS=Diagnostic Interview Schedule; DSM=Diagnostic and Statistical Manual of Mental Disorders; ICD=International Classification of Diseases; ISPI=Iowa Structured Psychiatric Interview; MD=mental disorder; MINI=Mini-International Neuropsychiatric Interview; NIMH-DIS=National Institute of Mental Health Diagnostic Interview Schedule; SADQ=Severity of Alcohol Dependence Questionnaire; SADS=Schedule for Affective Disorders and Schizophrenia; SCAN=Schedules for Clinical Assessment in Neuropsychiatry; SCID=Structured Clinical Interview for DSM Disorders; SMHWB=Survey of Mental Health and Wellbeing; SODQ=Severity of Dependence Questionnaire; SUD=substance use disorder; UK=United Kingdom; US=United Sates.

*Authors calculated prevalence of dual disorders.

†Authors provided additional information or clarification.

Supplementary Table 3: Race/ethnicity, nationality and country of birth in the included studies

Study	Country	Race/ethnicity, national identity and country of birth
Abram et al (2003)[3]	US	Race/ethnicity: African American (N=513), Non-Hispanic White (N=425), Hispanic (N=314)
Abram et al (1991)[4]	US	Race/ethnicity: Black (80.8%), White (12%), Hispanic (6.5%), Asian and American Indian (0.7%)
Alevizopoulos and Igoumenou (2016)[5]	Greece	NR
Andreoli et al (2014)[6]	Brazil	*(Race/ethnicity: "predominance of white prisoners born in the state")*
Assadi et al (2006)[7]	Iran	NR
Ayirolimeethal et al (2014)[8]	India	NR
Beaudette and Stewart (2016)[9]	Canada	Race/ethnicity: Aboriginal (20.7%), Black (8.9%), White (59.7%), Other (10.6%)
Bebbington et al (2017)[10]	UK	*(Race/ethnicity: "third of the prisoners in the current sample were black or black/white mixed")*
Benavides et al (2019)[11]	Ecuador	Country of birth: Place of family residence outside of the country (2.9%)
Blanchette and Motiuk (1996)[12]	Canada	Race/ethnicity: Caucasian (74.0%), Aboriginal (13.7%), Black (4.1%), Others (8.2%)
Brink et al (2001)[13]	Canada	Race/ethnicity: Caucasian (67.8%), Aboriginal (17.8%), Asian (4.5%), East Indian (3.5%), Black (3.5%), Other (3.0%)
Brown et al (2018a)[14]	Canada	Race/ethnicity: Indigenous (24.4%), Black (9.3%), White (53.5%), Other (10.5%), Missing (2.3%)
Brown et al (2018b)[15]	Canada	Race/ethnicity: Aboriginal (32.8%), Black (4.0%), White (57.0%); Other (7.3%)
Butler et al (2011)[16]	Australia	Race/ethnicity: Australian born (80%), Indigenous (19%)
Chiles et al (1990)[17]	US	NR
Cote and Hodgins (1990)[18]	Canada	NR
Curtin et al (2009)[19]	Ireland	Race/ethnicity: minority ethnicity (i.e. non-Caucasian, non-EU or Traveller) (19.8%)
Daniel et al (1988)[20]	US	Race/ethnicity: White (65%), Black (33%), Other (2%)
Denton (1995)[21]	Australia	Country of birth: Australian-born (85.7%), Overseas-born (14.3%)
Dudeck et al (2009)[22]	Germany	NR
Duffy et al (2006)[23]	Ireland	Race/ethnicity: White EU (88.2%), Black (0.35%), Chinese (0.3%), Asian (0.3%), Irish Traveller (10.7%), Other (0.3%)
Forry et al (2019)[24]	Uganda	NR
Fovet et al (2020)[25]	France	Nationality: *"majority of the sample was composed of French men"*
Gunter et al (2008)[26]	US	Race/ethnicity: African American (17.2%), Caucasian (71.6%), Other (11.3%)
Herrman et al (1991)[27]	Australia	NR
Indig et al (2016)[28]	New Zealand	Race/ethnicity: European (35.0%), Māori (51.5%), Pacific peoples (9.8%), Other/Not recorded (3.7%)<br>Country of birth: New Zealand (90.9%), Other (9.1%)
Joshi et al (2014)[29]	India	Nationality: Indian (84%), Foreign nationals (14%)
Linehan et al (2005)[30]	Ireland	Race/ethnicity: Caucasian (83.1%), Non EU European Caucasian (2.9%), African (5.7%), Chinese (1.8%), Irish Traveller (4.2%), Other (2.3%)
López et al (2016)[31]	Spain	Country of birth: Spain (81.8%)
Lukasiewicz et al (2009)[32]	France	NR
Lukasiewicz et al (2007)[33]	France	NR
Lynch et al (2014)[34]	US	Race/ethnicity: White or Caucasian (38%), African American or Black (37%), Latina (15%), American Indian (4%), Other (6%)
Mir et al (2015)[35]	Germany	Country of birth: Non-migrant (83%)
Mundt et al (2013)[36]	Chile	NR
Mundt and Baranyi (2020)[37]	Chile	Country of birth: Chilean (95.1%), Non-Chilean migrant populations (4.9%)
Nacher et al (2018)[38]	French Guiana	Country of birth: French Guiana (47.8%), other French territories (3%), Suriname (15.1%), Guyana (14.3%), Brazil (10.6%), Haiti (3.1%)
Naidoo and Mkize (2012)[39]	South Africa	Race/ethnicity: African (90.7%), Colored (3.1%), Indian (5.2%), White (0.5%), Other (0.5%)
Nanéma et al (2014)[40]	Burkina Faso	Nationality: Burkina Faso (93.8%); foreign nationals (6.2%)
Piselli et al (2015)[41]	Italy	Nationality: foreign nationals (33.8%)<br>Race/ethnicity: Italy (66.2%), Africa (12.5%), Eastern Europe (16.2%), Other (5.1%)
Piselli et al (2009)[42]	Italy	Race/ethnicity: Southern-European (37.0%), African (33.3%), Eastern-European (29.7%)
Proctor et al (2019)[43]	US	Race/ethnicity: White (86.0%), American Indian (7.5%), Black (3.5%)
Schröder (2005)[44]	Germany	Nationality: German (93.4%), Other Europeans (5.3%), Other Nationalities (1.3%)<br>Country of birth: German (72.4%), Migrant (27.6%)
Simpson et al (1999)[45]	New Zealand	Race/ethnicity: Maori (48.4%), Pacific peoples (8.3%), Pakeha (30.9%), Other (12.3%)
Tye and Mullen (2006)[46]	Australia	Race/ethnicity: Aboriginal (9.7%)

Vicens et al (2011)[47]	Spain	Country of birth: Spain (72.5%), Africa (9.9%), America (8.9%), Asia (1.7%), Europe (6.2%), Other (0.1%), Unknown (0.6%)
Widmann (2006)[48]	Germany	Nationality: German (84.1%), Other Europeans (4.8%), Other Nationalities (11.1%)
Wright et al (2006)[49] A	Ireland	Nationality: Irish origin (82.3%)
Zabala-Baños et al (2016)[50]	Spain	Country of birth: Spain (54.9%), Africa (7.6%), South America (26.6%), Asia (0.5%), Europe (10.3%)
Zamzam and Hatta (2000)[51]	Malaysia	Race/ethnicity: Malay (76.3%), Indians (12.5%), Chinese (8.8%) Kadazans (2.5%)
Zhong et al (2020)[52]	China	Race/ethnicity: Han (90.8%), Tujia ethnicity (3.6%), Miao ethnicity (2.5%), Others (3.1%)

Note: Categories and groups are reported as indicated in the studies. NR=not reported, UK=United Kingdom; US=United Sates.

Supplementary Table 4: Quality appraisal of included samples

Study	1	2	3	4	5	6	7	8	9	10	SCORE
Abram et al (2003)[3]	0	1	1	1	0	1	1	0	0	1	6
Abram et al (1991)[4]	0	1	1	1	1	1	1	1	0	1	8
Alevizopoulos and Igoumenou (2016)[5]	0	1	1	1	1	1	1	0	1	1	8
Andreoli et al (2014)[6] M	1	1	1	0	1	1	1	0	0	1	7
Andreoli et al (2014)[6] F	1	1	1	1	1	1	1	0	0	1	8
Assadi et al (2006)[7]	0	1	1	1	1	1	1	1	1	1	9
Ayirolimeethal et al (2014)[8]	0	1	1	1	1	1	1	1	0	0	7
Beaudette and Stewart (2016)[9]	1	1	1	1	1	1	1	0	1	1	9
Bebbington et al (2017)[10] M	0	1	1	0	1	1	1	1	1	1	8
Bebbington et al (2017)[10] F	0	1	1	0	1	1	1	1	1	1	8
Benavides et al (2019)[11]	0	1	1	1	1	1	1	1	1	1	9
Blanchette and Motiuk (1996)[12]	0	1	0	1	1	1	1	NR	0	1	6
Brink et al (2001)[13]	0	1	1	1	1	1	1	1	1	1	9
Brown et al (2018a)[14]	1	1	0	0	1	1	1	0	1	1	7
Brown et al (2018b)[15]	1	1	0	0	1	1	1	0	1	1	7
Butler et al (2011)[16] M	1	1	1	NR	1	1	1	0	0	1	7
Butler et al (2011)[16] F	1	1	1	NR	1	1	1	0	0	1	7
Chiles et al (1990)[17]	0	NR	0	NR	0	1	1	NR	1	1	4
Cote and Hodgins (1990)[18]	1	1	1	1	0	1	1	0	1	1	8
Curtin et al (2009)[19]	1	1	1	1	1	1	1	1	1	1	10
Daniel et al (1988)[20]	0	1	0	1	1	1	1	1	1	1	8
Denton (1995)[21]	0	1	0	1	1	1	1	0	1	1	7
Dudeck et al (2009)[22]	0	1	0	1	1	1	1	0	1	1	7
Duffy et al (2006)[23]	1	1	1	1	1	1	1	0	1	1	9
Forry et al (2019)[24]	1	1	1	1	1	1	1	0	1	0	8
Fovet et al (2020)[25] M	1	1	1	0	1	1	1	1	1	0	8
Fovet et al (2020)[25] F	1	1	0	0	1	1	1	1	1	0	7
Gunter et al (2008)[26] M	0	1	1	NR	1	1	1	0	1	1	7
Gunter et al (2008)[26] F	0	1	0	NR	1	1	1	0	1	1	6
Herrman et al (1991)[27] M	0	1	0	0	1	1	1	1	1	1	7
Herrman et al (1991)[27] F	0	1	0	0	1	1	1	1	1	1	7
Indig et al (2016)[28] M	1	1	1	1	1	1	1	0	1	1	9
Indig et al (2016)[28] F	1	1	0	0	1	1	1	0	1	1	7
Joshi et al (2014)[29]	0	1	0	NR	1	0	1	1	0	1	5
Linehan et al (2005)[30]	1	1	1	1	1	1	1	0	1	1	9
López et al (2016)[31]	1	1	1	1	1	1	1	1	1	1	10
Lukasiewicz et al (2009)[32*]	1	1	1	0	1	1	1	1	1	0	8
Lukasiewicz et al (2007)[33*]	1	1	1	0	1	1	1	1	0	0	7
Lynch et al (2014)[34]	0	1	1	1	1	1	1	0	1	1	8
Mir et al (2015)[35]	0	1	0	1	1	1	1	1	1	1	8
Mundt et al (2013)[36] M	1	1	1	1	1	1	1	0	1	1	9
Mundt et al (2013)[36] F	1	1	0	1	1	1	1	0	1	1	8
Mundt and Baranyi (2020)[37] M	0	1	1	1	1	1	1	1	1	1	9
Mundt and Baranyi (2020)[37] F	0	1	0	1	1	1	1	1	1	1	8
Nacher et al (2018)[38]	1	1	1	1	1	1	1	1	1	0	9
Naidoo and Mkize (2012)[39]	0	1	0	1	1	1	1	1	1	0	7
Nanéma et al (2014)[40]	0	1	1	1	1	1	1	0	1	0	7
Piselli et al (2015)[41]	0	1	1	1	1	1	1	1	1	1	9
Piselli et al (2009)[42]	0	1	1	0	1	1	1	1	1	1	8
Proctor et al (2019)[43]	0	1	1	NR	1	1	1	0	0	1	6
Schröder (2005)[44]	0	1	0	0	1	1	1	1	0	1	6
Simpson et al (1999)[45]	1	1	1	1	1	1	1	0	1	0	8
Tye and Mullen (2006)[46]	1	1	0	1	1	1	1	1	1	1	9
Vicens et al (2011)[47]	0	1	1	1	1	1	1	1	0	1	8
Widmann (2006)[48]	0	1	0	0	1	1	1	1	1	1	7
Wright et al (2006)[49] A	1	1	0	0	1	1	1	1	1	1	8
Wright et al (2006)[49] C	1	1	0	1	1	1	1	1	1	1	9
Zabala-Baños et al (2016)[50]	0	1	0	1	1	1	1	1	1	1	8
Zamzam and Hatta (2000)[51]	0	1	0	1	1	1	1	1	0	1	7
Zhong et al (2020)[52]	1	1	1	1	1	1	1	0	1	1	9

A=admission sample; C=cross-sectional sample; F=female; M=male; NR=not reported.
*Studies report on the same sample.

Table 5: Prevalence estimates and odds ratios of co-occurring mental and substance use disorders in prison populations

	Prevalence						Odds Ratio		
	Range	Estimate	95% CI	I^2	% of rMD	% of rSUD	Estimate	95% CI	I^2
**Current prevalence**									
Non-affective psychosis									
& Substance use disorders (n=11236; k=28)	0·0-14·6	3·5	2·2-5·0	90·9	49·2	9·2	1·7	1·4-2·2	34·6
& Alcohol use disorders (n=11669; k=26)	0·0-8·7	1·8	1·1-2·7	82·4	28·3	10·7	2·0	1·5-2·6	49·6
& Drug use disorders (n=10839; k=24)	0·0-16·8	2·4	1·2-4·0	92·8	37·0	10·1	2·0	1·4-2·9	68·3
Depression									
& Substance use disorders (n=11133; k=27)	0·8-49·8	9·1	5·6-13·3	96·9	51·6	23·3	1·6	1·3-2·0	53·0
& Alcohol use disorders (n=13528; k=29)	0·2-30·4	5·1	3·1-7·7	95·6	26·2	27·4	1·8	1·5-2·3	49·5
& Drug use disorders (n=11991; k=26)	0·0-44·5	5·4	2·7-8·9	97·5	34·1	25·8	1·8	1·4-2·2	42·6
Axis I disorders									
& Substance use disorders (n=10998; k=24)	1·1-57·6	20·7	13·8-28·5	98·4	51·4	47·9	2·0*	1·4-2·9	79·8
& Alcohol use disorders (n=8809; k=18)	1·3-34·8	11·1	6·6-16·5	97·0	30·3	49·1	2·3	1·8-3·1	62·1
& Drug use disorders (n=7640; k=17)	2·1-50·2	15·8	8·85-24·2	98·2	43·2	47·2	2·6	2·1-3·3	46·7
**Lifetime prevalence**									
Non-affective psychosis									
& Substance use disorders (n=7241; k=21)	0·0-28·3	6·9	4·7-9·4	89·1	86·0	9·8	1·7	1·1-2·5	41·9
& Alcohol use disorders (n=7119; k=14)	0·0-19·0	4·9	2·4-8·0	96·5	60·5	11·0	2·0	1·6-2·6	0·0
& Drug use disorders (n=6956; k=12)	0·0-26·1	5·3	2·3-9·4	95·4	63·1	10·5	2·1	1·7-2·6	0·0
Major depression									
& Substance use disorders (n=6485; k=18)	6·9-42·9	22·2	16·9-28·0	94·7	60·0	20·7	1·4*	1·1-1·8	57·1
& Alcohol use disorders (n=8828; k=15)	2·0-36·8	12·4	7·9-17·8	97·0	32·4	25·4	1·7	1·2-2·4	77·3
& Drug use disorders (n=8845; k=15)	3·0-35·7	14·3	9·5-19·8	95·0	43·9	30·4	2·0	1·3-2·9	93·2
Axis I disorders									
& Substance use disorders (n=5002; k=11)	14·3-68·8	39·9	28·3-52·1	96·8	89·0	53·7	2·7*	1·8-4·0	72·9
& Alcohol use disorders (n=3884; k=8)	7·6-52·2	27·9	16·0-41·7	98·0	67·5	45·4	2·3	1·4-3·5	57·6
& Drug use disorders (n=3884; k=8)	5·1-64·9	29·7	14·3-47·9	98·6	68·2	52·4	2·7	2·0-3·6	52·2

rMD=reference mental disorder; rSUD=reference substance use disorder; n=pooled sample size; k=number of included samples.
*Due to missing reference values, odds ratios could not be calculated for all included samples.

Supplementary Table 6: Meta-regression exploring heterogeneity across estimates of comorbid non-affective psychosis and substance use disorders

	Substance use disorders				Alcohol use disorders				Drug use disorders			
	b	SE	p	p-adj*	b	SE	p	p-adj*	b	SE	p	p-adj*
Current prevalence												
**Univariate**	k=28				k=26				k=24			
Sex (ref female)	-0·021	0·039	0·588	0·840	0·027	0·028	0·349	0·628	0·032	0·045	0·486	0·912
Sample size (ref n>200)	0·002	0·037	0·956	0·965	-0·016	0·028	0·571	0·919	-0·027	0·044	0·547	0·912
Average age	-0·004	0·005	0·462	0·770	0·000	0·004	0·936	0·936	-0·000	0·007	0·992	0·992
Year of data collection	-0·002	0·003	0·429	0·770	0·001	0·002	0·643	0·919	0·002	0·002	0·361	0·912
Non-response rate	0·002	0·001	0·159	0·770	0·002	0·001	0·038	0·380	0·002	0·002	0·293	0·912
Type of recruitment (ref admission)	-0·036	0·037	0·341	0·770	-0·033	0·028	0·252	0·683	-0·027	0·042	0·529	0·912
Country classification (ref HIC)	-0·004	0·043	0·930	0·965	0·010	0·033	0·773	0·936	0·053	0·046	0·262	0·912
Diagnostic classification (ref DSM)	0·073	0·042	0·094	0·770	0·046	0·028	0·116	0·580	-0·018	0·059	0·765	0·937
% of the sample with previous imprisonment	0·000	0·001	0·965	0·965	-0·001	0·001	0·273	0·683	0·000	0·001	0·843	0·937
Data source (ref unpublished)	0·031	0·037	0·398	0·770	-0·005	0·030	0·883	0·936	-0·012	0·050	0·814	0·937
**Multivariate**												
NA												
Lifetime prevalence												
**Univariate**	k=21				k=14				k=12			
Sex (ref female)	-0·011	0·048	0·822	0·822	0·025	0·060	0·682	0·801	0·010	0·076	0·903	0·988
Sample size (ref n>200)	0·014	0·044	0·749	0·822	0·048	0·060	0·440	0·801	0·072	0·071	0·334	0·601
Average age	0·016	0·008	0·068	0·680	0·002	0·009	0·801	0·801	0·012	0·011	0·313	0·601
Year of data collection	0·003	0·003	0·229	0·822	0·001	0·003	0·785	0·801	0·002	0·003	0·509	0·764
Non-response rate	-0·001	0·002	0·682	0·822	0·001	0·002	0·790	0·801	0·000	0·003	0·921	0·988
Type of recruitment (ref admission)	-0·013	0·045	0·768	0·822	-0·034	0·059	0·582	0·801	0·001	0·074	0·988	0·988
Country classification (ref HIC)	-0·079	0·096	0·425	0·822	-0·185	0·095	0·074	0·549	-0·132	0·122	0·304	0·601
Diagnostic classification (ref DSM)	-0·026	0·051	0·616	0·822	NA							
% of the sample with previous imprisonment	-0·001	0·002	0·651	0·822	0·006	0·002	0·122	0·549	0·005	0·004	0·310	0·601
Data source (ref unpublished)	-0·038	0·045	0·409	0·822	-0·057	0·058	0·345	0·801	-0.080	0.071	0.288	0·601
**Multivariate**												
NA												

Bold typeface denotes false discovery rate-corrected significance. b=unstandardized coefficient, DSM= Diagnostic and Statistical Manual of Mental Disorders, HIC=high-income countries, k=number of included samples, n=sample size, NA=not applicable, SE=standard error.
* False discovery rate adjusted p-value.

Supplementary Table 7: Meta-regression exploring heterogeneity across estimates of comorbid depression and substance use disorders

	Substance use disorders				Alcohol use disorders				Drug use disorders			
	b	SE	p	p-adj*	b	SE	p	p-adj*	b	SE	p	p-adj*
Current prevalence												
**Univariate**	k=27				k=29				k=26			
Sex (ref female)	-0·028	0·068	0·683	0·946	-0·026	0·052	0·623	0·759	-0·007	0·068	0·919	0·919
Sample size (ref n>200)	-0·005	0·066	0·946	0·946	0·057	0·051	0·273	0·546	-0·016	0·069	0·823	0·919
Average age	-0·010	0·010	0·333	0·946	-0·003	0·009	0·732	0·759	-0·002	0·012	0·850	0·919
Year of data collection	0·004	0·005	0·388	0·946	0·004	0·004	0·266	0·546	0·006	0·005	0·208	0·520
Non-response rate	0·003	0·002	0·164	0·820	0·006	0·002	0·014	0·140	0·004	0·003	0·173	0·520
Type of recruitment (ref admission)	-0·172	0·059	0·008	0·080	-0·045	0·053	0·399	0·643	-0·099	0·064	0·135	0·520
Country classification (ref HIC)	0·013	0·078	0·865	0·946	-0·043	0·055	0·450	0·643	0·021	0·070	0·763	0·919
Diagnostic classification (ref DSM)	0·044	0·079	0·584	0·946	0·063	0·052	0·236	0·546	-0·114	0·075	0·145	0·520
% of the sample with previous imprisonment	0·001	0·003	0·842	0·946	0·001	0·002	0·759	0·759	0·003	0·003	0·330	0·660
Data source (ref unpublished)	0·033	0·067	0·625	0·946	-0·063	0·052	0·238	0·546	-0.063	0.074	0.402	0·670
**Multivariate**												
NA												
Lifetime prevalence												
**Univariate**	k=18				k=15				k=15			
Sex (ref female)	-0·026	0·071	0·716	0·895	-0·061	0·070	0·396	0·792	-0·088	0·067	0·209	0·418
Sample size (ref n>200)	-0·008	0·066	0·906	0·906	0·137	0·060	0·041	0·190	0·125	0·061	0·063	0·315
Average age	0·020	0·013	0·137	0·457	0·003	0·011	0·831	0·940	0·015	0·010	0·167	0·418
Year of data collection	0·001	0·004	0·843	0·906	-0·0003	0·004	0·930	0·940	0·003	0·003	0·373	0·554
Non-response rate	0·003	0·002	0·263	0·526	**0·007**	**0·002**	**0·004**	**0·040**	0·006	0·002	0·022	0·220
Type of recruitment (ref admission)	0·098	0·060	0·122	0·456	0·006	0·073	0·940	0·940	0·066	0·074	0·388	0·554
Country classification (ref HIC)	0·206	0·127	0·122	0·456	-0·142	0·077	0·090	0·225	-0·048	0·078	0·546	0·607
Diagnostic classification (ref DSM)	-0·160	0·130	0·234	0·526	-0·067	0·101	0·520	0·833	0·011	0·105	0·917	0·917
% of the sample with previous imprisonment	-0·001	0·003	0·644	0·895	0·007	0·003	0·057	0·190	0·004	0·002	0·140	0·418
Data source (ref unpublished)	0·050	0·065	0·456	0·760	-0·040	0·071	0·583	0·833	-0·049	0·069	0·494	0·607
**Multivariate**												
NA												

Bold typeface denotes false discovery rate-corrected significance. b=unstandardized coefficient, DSM= Diagnostic and Statistical Manual of Mental Disorders, HIC=high-income countries, k=number of included samples, n=sample size, NA=not applicable, SE=standard error.
* False discovery rate adjusted p-value.

Supplementary Table 8: Meta-regression exploring heterogeneity across estimates of comorbid Axis I and substance use disorders

	Substance use disorders				Alcohol use disorders				Drug use disorders			
	b	SE	p	p-adj*	b	SE	p	p-adj*	b	SE	p	p-adj*
Current prevalence												
**Univariate**	k=24				k=18				k=17			
Sex (ref female)	-0·080	0·088	0·376	0·583	0·004	0·077	0·955	0·973	-0·006	0·104	0·955	0·994
Sample size (ref n>200)	0·031	0·093	0·742	0·742	-0·003	0·079	0·973	0·973	-0·015	0·104	0·887	0·994
Average age	-0·012	0·016	0·467	0·584	0·009	0·017	0·623	0·853	0·010	0·026	0·724	0·994
Year of data collection	0·006	0·006	0·346	0·584	0·008	0·004	0·050	0·167	0·011	0·005	0·035	0·260
Non-response rate	**0·009**	**0·003**	**0·010**	**0·050**	0·007	0·003	0·025	0·125	0·006	0·004	0·153	0·382
Type of recruitment (ref admission)	**-0·266**	**0·074**	**0·002**	**0·020**	-0·228	0·075	0·008	0·080	-0·207	0·098	0·052	0·260
Country classification (ref HIC)	0·106	0·133	0·435	0·584	0·042	0·101	0·682	0·853	0·148	0·114	0·213	0·426
Diagnostic classification (ref DSM)	0·122	0·096	0·221	0·584	0·075	0·084	0·386	0·772	-0·091	0·134	0·507	0·845
% of the sample with previous imprisonment	-0·002	0·004	0·673	0·742	-0·002	0·004	0·627	0·853	0·000	0·004	0·994	0·994
Data source (ref unpublished)	-0·083	0·091	0·368	0·584	-0·102	0·087	0·257	0·640	-0·226	0·145	0·138	0·383
**Multivariate**												
Non-response rate	**0.007**	**0.003**	**0.043**									
Type of recruitment (ref admission)	**-0.179**	**0.075**	**0.029**									
Lifetime prevalence												
**Univariate**	k=11				k=8				k=8			
Sex (ref female)	-0·095	0·112	0·419	0·629	NA				NA			
Sample size (ref n>200)	0·043	0·115	0·716	0·806								
Average age	0·036	0·024	0·182	0·349								
Year of data collection	**0·019**	**0·006**	**0·010**	**0·045**								
Non-response rate	**0·011**	**0·003**	**0·007**	**0·045**								
Type of recruitment (ref admission)	-0·010	0·116	0·936	0·936								
Country classification (ref HIC)	NA											
Diagnostic classification (ref DSM)	-0·271	0·175	0·157	0·349								
% of the sample with previous imprisonment	-0·007	0·011	0·578	0·743								
Data source (ref unpublished)	-0·152	0·108	0·194	0·349								
**Multivariate**												
NA												

Bold typeface denotes false discovery rate-corrected significance. b=unstandardized coefficient, DSM= Diagnostic and Statistical Manual of Mental Disorders, HIC=high-income countries, k=number of included samples, n=sample size, NA=not applicable, SE=standard error.

* False discovery rate adjusted p-value.

Supplementary Table 9: Prevalence estimates and odds ratios of comorbid mental and substance use disorders applying narrow disorder criteria for non-affective psychosis and major depression

		Prevalence						Odds Ratio		
		Range	Estimate	95% CI	I^2	% of rMD	% of rSUD	Estimate	95% CI	I^2
	Current prevalence									
Non-affective psychosis										
&	Substance use disorders (n=9967; k=27)	0-14·6	3·6	2·2-5·2	91·0	48·3	10·1	1·7	1·3-2·2	35·9
&	Alcohol use disorders (n=10400; k=25)	0-8·7	1·9	1·1-2·7	82·9	27·3	11·0	1·8	1·4-2·4	42·7
&	Drug use disorders (n=9570; k=23)	0-16·8	2·5	1··2-4·1	93·1	36·2	11·7	2·1	1·4-3·0	69·4
Major depression										
&	Substance use disorders (n=7560; k=19)	0·8-49·8	10·1	5·4-16·0	97·1	53·1	21·7	1·7	1·3-2·2	52·3
&	Alcohol use disorders (n=8436; k=20)	0·2-30·4	4·9	2·6-7·7	94·0	29·2	22·2	1·7	1·2-2·3	56·4
&	Drug use disorders (n= 8745; k=21)	0·7-44·5	6·6	3·3-10·9	97·0	40·1	23·4	1·8	1·4-2·2	38·6
	Lifetime prevalence									
Non-affective psychosis										
&	Substance use disorders (n=5770; k=16)	0-28·3	7·2	4·4-10·5	90·9	88·5	10·3	1·9	1·2-3·0	32·4
&	Alcohol use disorders (n=7119; k=14)	0-19·0	4·9	2·4-8·0	96·5	60·5	11·0	2·0	1·6-2·6	0·0
&	Drug use disorders (n=6956; k=12)	0-26·1	5·3	2·3-9·4	95·4	63·1	10·5	2·1	1·7-2·6	0·0
Major depression										
&	Substance use disorders (n=4118; k=9)	6·9-38·3	19·0	12·2-26·9	90·1	84·3	23·8	1·6*	1·3-1·9	0·0
&	Alcohol use disorders (n=3993; k=9)	4·1-25·3	12·7	7·3-22·3	93·8	68·3	22·7	1·7	1·1-2·5	44·4
&	Drug use disorders (n=4073; k=10)	3·0-35·7	12·6	6·9-19·7	96·0	66·4	22·1	1·7	1·4-2·1	0·0

rMD=reference mental disorder; rSUD=reference substance use disorder; n=pooled sample size; k=number of included samples.

*Due to missing reference values, odds ratios could not be calculated for all included samples.

Supplementary Table 10: Egger's test indicating funnel plot asymmetry

	Egger's test			Egger's test after exclusion of influential sample(s)		
	bias	SE of bias	p-value	bias	SE of bias	p-value
**Current prevalence**						
Non-affective psychosis						
& Substance use disorders	0·917	1·392	0·515	NA		
& Alcohol use disorders	0·549	1·115	0·627	NA		
& Drug use disorders	1·299	1·858	0·492	NA		
Depression						
& Substance use disorders	1·162	2·457	0·640	NA		
& Alcohol use disorders	3·256	1·978	0·111	NA		
& Drug use disorders	2·225	2·933	0·455	NA		
Axis I disorders						
& Substance use disorders	2·875	3·742	0·451	NA		
& Alcohol use disorders	2·911	3·007	0·347	NA		
& Drug use disorders	4·514	3·974	0·274	NA		
**Lifetime prevalence**						
Non-affective psychosis						
& Substance use disorders	0·056	1·408	0·969	NA		
& Alcohol use disorders	3·914	2·394	0·128	NA		
& Drug use disorders	3·662	2·325	0·146	NA		
Depression						
& Substance use disorders	1·510	2·084	0·479	NA		
& Alcohol use disorders	**6·445**	**2·348**	**0·017**	3·125	2·007	0·145
& Drug use disorders	3·807	2·040	0·085	NA		
Axis I disorders						
& Substance use disorders	0·237	4·137	0·956	NA		
& Alcohol use disorders	NA					
& Drug use disorders	NA					

SE=standard error.

Supplementary Figure 1: Funnel plots for co-occuring substance use disorders and (1) non-affective psychosis, (2) depression and (3) Axis I disorders

(1)

Current prevalence | Lifetime prevalence

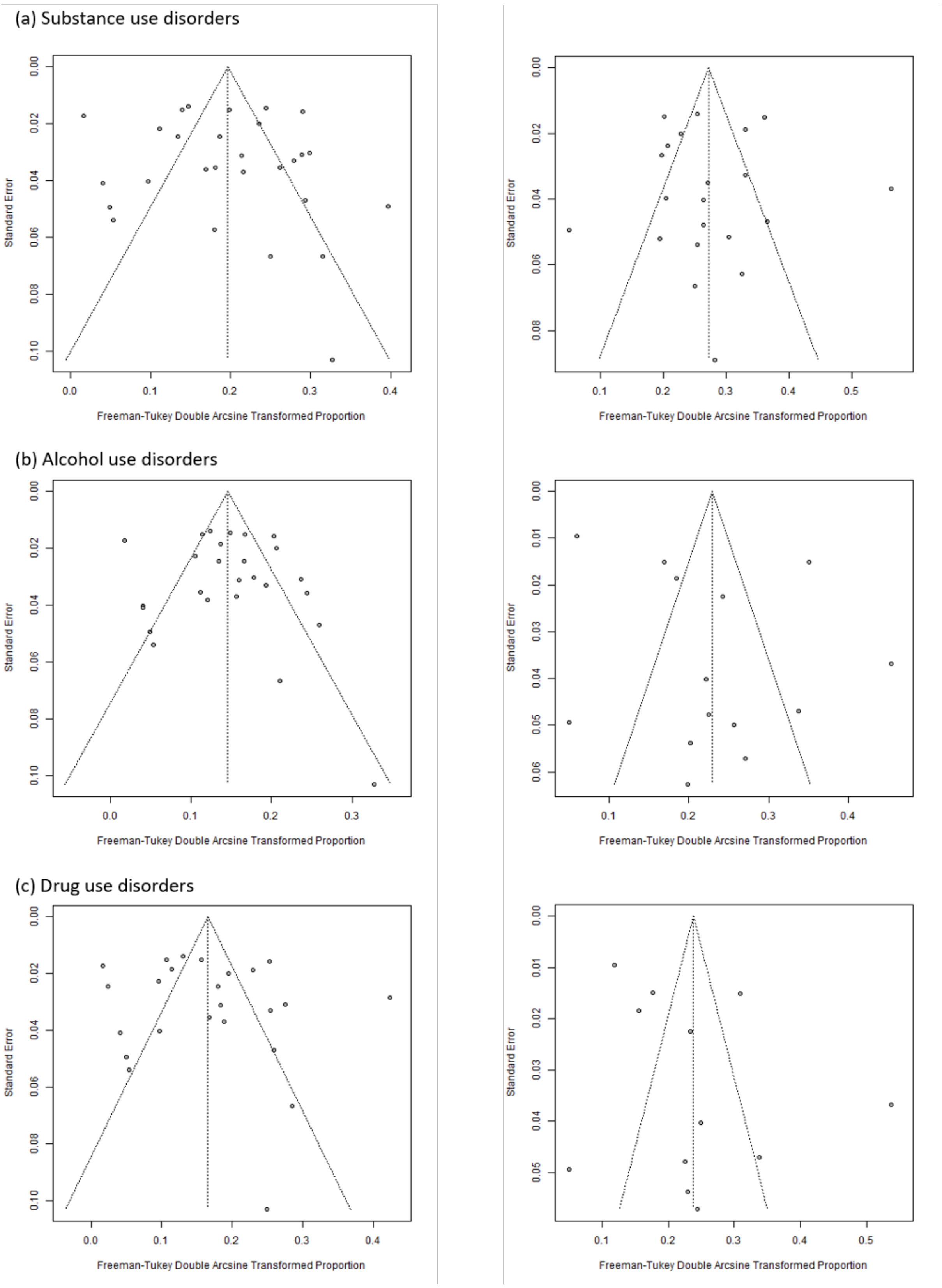

(2)

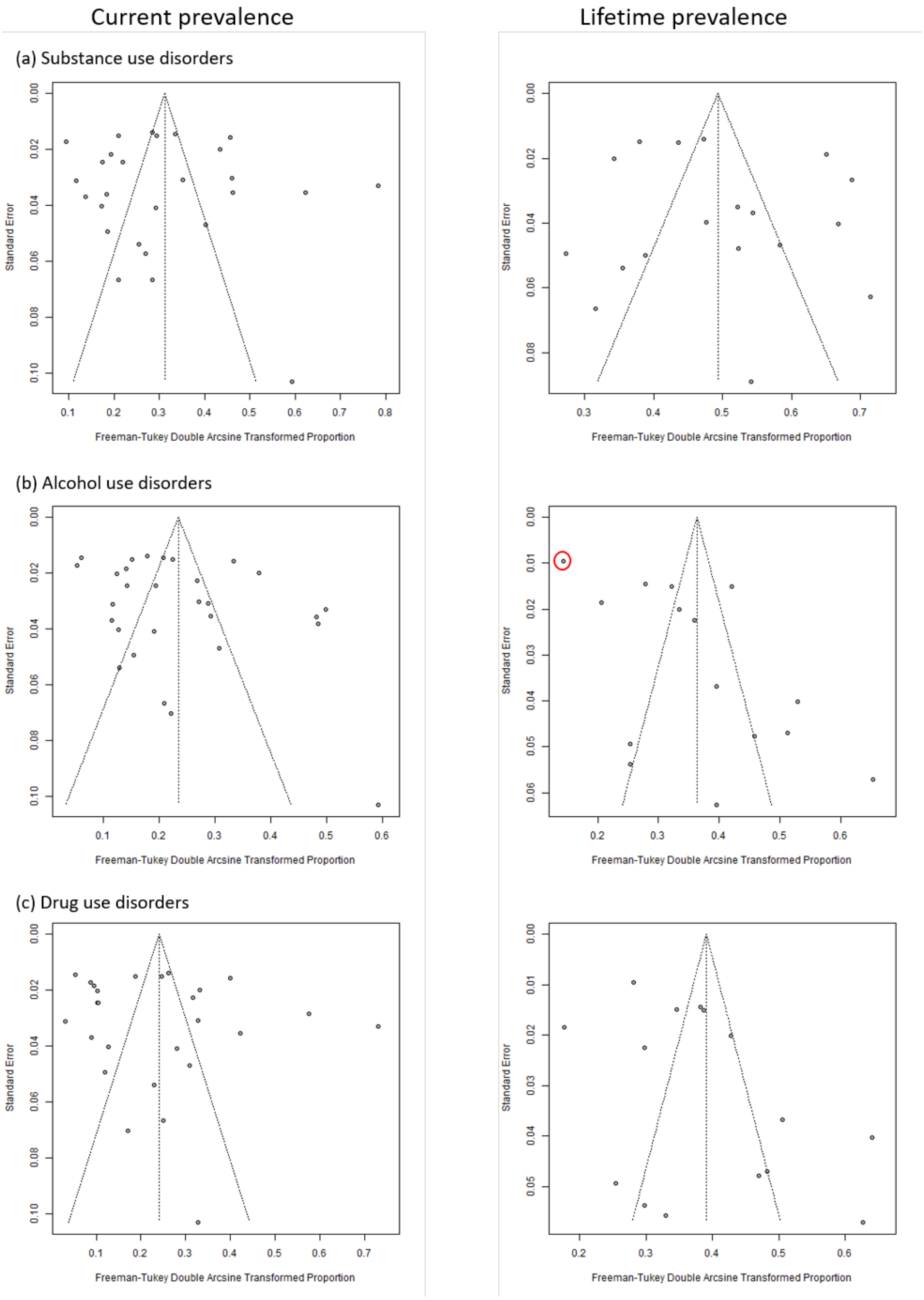

Red-circled estimate was excluded for sensitivity (see Supplementary Table 6).

(3)

Current prevalence

Lifetime prevalence

(a) Substance use disorders

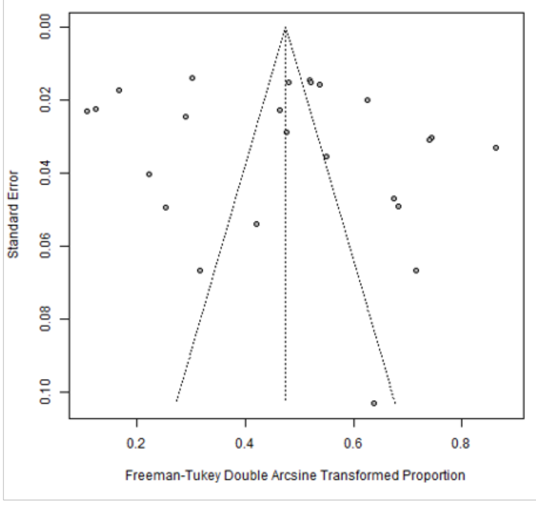

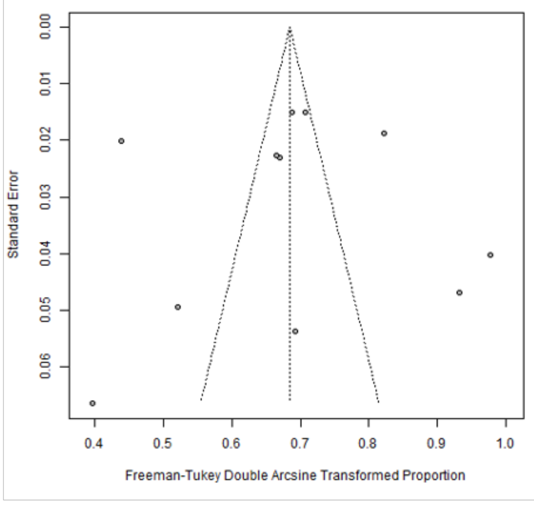

(b) Alcohol use disorders

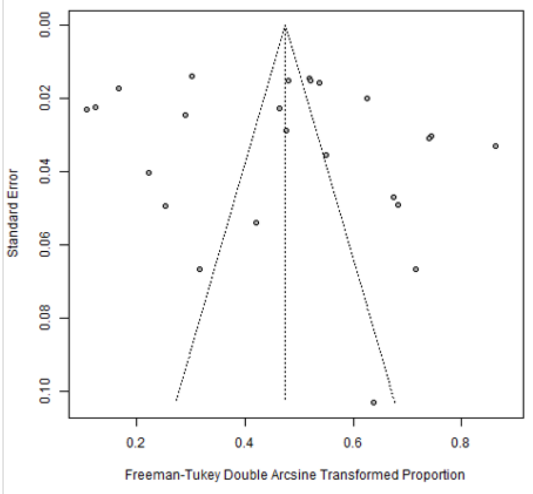

(c) Drug use disorders

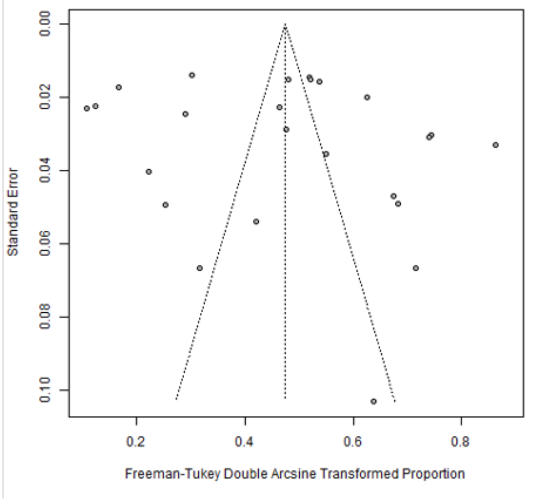

Supplementary Table 11: Meta-regression with quality appraisal score

		Quality appraisal score		
		b	SE	p
	**Current prevalence**			
Non-affective psychosis				
&	Substance use disorders	-0·016	0·018	0·372
&	Alcohol use disorders	-0·021	0·015	0·186
&	Drug use disorders	-0·004	0·023	0·853
Depression				
&	Substance use disorders	-0·009	0·034	0·798
&	Alcohol use disorders	0·011	0·027	0·691
&	Drug use disorders	0·041	0·033	0·224
Axis I disorders				
&	Substance use disorders	-0·034	0·044	0·447
&	Alcohol use disorders	-0·005	0·040	0·909
&	Drug use disorders	0·014	0·050	0·777
	**Lifetime prevalence**			
Non-affective psychosis				
&	Substance use disorders	-0·001	0·017	0·955
&	Alcohol use disorders	-0·002	0·023	0·921
&	Drug use disorders	-0·0001	0·027	0·998
Depression				
&	Substance use disorders	-0·012	0·025	0·619
&	Alcohol use disorders	-0·049	0·025	0·075
&	Drug use disorders	-0·041	0·026	0·142
Axis I disorders				
&	Substance use disorders	-0·042	0·048	0·401
&	Alcohol use disorders	NA		
&	Drug use disorders	NA		

b=unstandardized coefficient, SE=standard error.

Supplementary Table 12: Prevalence of comorbid mental and substance use disorders before and after exclusion of outlier estimates

		Number of included samples	Prevalence					
			Before exclusion			After exclusion		
			Estimate	95% CI	I^2	Estimate	95% CI	I^2
	**Current prevalence**							
Non-affective psychosis								
&	Substance use disorders	20 out of 28	3·5	2·2-5·0	90·9	4·0	2·9-5·2	77·3
&	Alcohol use disorders	23 out of 26	1·8	1·1-2·7	82·4	1·8	1·2-2·5	68·6
&	Drug use disorders	18 out of 24	2·4	1·2-4·0	92·8	2·3	1·4-3·4	77·6
Depression								
&	Substance use disorders	14 out of 27	9·1	5·6-13·3	96·9	6·9	5·0-9·0	75·0
&	Alcohol use disorders	17 out of 29	5·1	3·1-7·7	95·6	4·0	2·9-5·4	70·9
&	Drug use disorders	14 out of 26	5·4	2·7-8·9	97·5	5·9	4·0-8·0	81·4
Axis I disorders								
&	Substance use disorders	10 out of 24	20·7	13·8-28·5	98·4	22·7	19·5-26·0	67·1
&	Alcohol use disorders	10 out of 18	11·1	6·6-16·5	97·0	10·5	6·8-14·9	84·5
&	Drug use disorders	7 out of 17	15·8	8·85-24·2	98·2	19·3	14·7-24·3	77·5
	**Lifetime prevalence**							
Non-affective psychosis								
&	Substance use disorders	18 out of 21	6·9	4·7-9·4	89·1	6·1	2·1-6·8	69·0
&	Alcohol use disorders	10 out of 14	4·9	2·4-8·0	96·5	4·5	3·1-6·1	55·6
&	Drug use disorders	9 out of 12	5·3	2·3-9·4	95·4	5·2	3·3-7·5	87·0
Major depression								
&	Substance use disorders	11 out of 18	22·2	16·9-28·0	94·7	20·4	16·3-24·8	68·2
&	Alcohol use disorders	11 out of 15	12·4	7·91-17·8	97·0	12·2	8·9-16·0	86·8
&	Drug use disorders	11 out of 15	14·3	9·54-19·8	95·0	13·6	10·1-17·6	81·6
Axis I disorders								
&	Substance use disorders	7 out of 11	39·9	28·3-52·1	96·8	40·1	32·5-48·0	89·8
&	Alcohol use disorders	6 out of 8	27·9	16·0-41·7	98·0	28·7	18·3-40·4	94·7
&	Drug use disorders	6 out of 8	29·7	14·3-47·9	98·6	29·6	17·3-43·7	94·8

References

1. Munn Z, Moola S, Riitano D, Lisy K. The development of a critical appraisal tool for use in systematic reviews addressing questions of prevalence. *Int J Health Policy Manag* 2014; **3**: 123–8.

2. Hoy D, Brooks P, Woolf A, et al. Assessing risk of bias in prevalence studies: modification of an existing tool and evidence of interrater agreement. *J Clin Epidemiol* 2012; **65**: 934–9.

3. Abram KM, McClelland GM, Teplin LA. Comorbidity of severe psychiatric disorders and substance use disorders among women in jail. *Am J Psychiatry* 2003; **160**: 1007–10.

4. Abram KM, Teplin LA. Co-occurring disorders among mentally ill jail detainees: Implications for public policy. *Am Psychol* 1991; **46**: 1036–45.

5. Alevizopoulos G, Igoumenou A. Psychiatric disorders and criminal history in male prisoners in Greece. *Int J Law Psychiatry* 2016; **47**: 171–5.

6. Andreoli SB, Santos MMd, Quintana MI, et al. Prevalence of mental disorders among prisoners in the State of Sao Paulo, Brazil. *PLoS ONE* 2014; **9**: e88836.

7. Assadi SM, Maryam N, Mahdi P, et al. Psychiatric morbidity among sentenced prisoners: prevalence study in Iran. *Br J Psychiatry* 2006; **188**: 159–64.

8. Ayirolimeethal A, Ragesh G, Ramanujam JM, George B. Psychiatric morbidity among prisoners. *Indian J Psychiatry* 2014; **56**: 150–53.

9. Beaudette JN, Stewart LA. National Prevalence of Mental Disorders among Incoming Canadian Male Offenders. *Can J Psychiatry* 2016; **61**: 624–32.

10. Bebbington P, Jakobowitz S, McKenzie N, et al. Assessing needs for psychiatric treatment in prisoners: 1. Prevalence of disorder. *Soc Psychiatry Psychiatr Epidemiol* 2017; **52**: 221–9.

11. Benavides A, Chuchuca J, Klaic D, Waters W, Martin M, Romero-Sandoval N. Depression and psychosis related to the absence of visitors and consumption of drugs in male prisoners in Ecuador: A cross sectional study. *BMC Psychiatry* 2019; **19**: 248.

12. Blanchette K, Motiuk LL. Female offenders with and without major mental health problems: a comparative investigation. Ottawa, ON, Canada: Correctional Services Canada, 1996.

13. Brink JH, Doherty D, Boer A. Mental disorder in federal offenders: a Canadian prevalence study. *Int J Law Psychiatry* 2001; **24**: 339–56.

14. Brown G, Barker J, McMillan K, et al. Prevalence of Mental Disorder among Federally Sentenced Women Offenders: In-Custody and Intake Samples. Ottawa, ON, Canada: Correctional Service of Canada, 2018.

15. Brown GP, Barker J, McMillan K, Norman R, Derkzen D, Stewart LA. National Prevalence of Mental Disorders among Federally Sentenced Women Offenders: In Custody Sample. Ottawa, ON, Canada: Correctional Service of Canada, 2018.

16. Butler T, Indig D, Allnutt S, Mamoon H. Co-occurring mental illness and substance use disorder among Australian prisoners. *Drug Alcohol Rev* 2011; **30**: 188–94.

17. Chiles JA, von Cleve E, Jemelka RP, Trupin EW. Substance abuse and psychiatric disorders in prison inmates. *Hosp Community Psychiatry* 1990; **41**: 1132–4.

18. Cote G, Hodgins S. Co-occurring mental disorders among criminal offenders. *Bull Am Acad Psychiatry Law* 1990; **18**: 271–81.

19. Curtin K, Monks S, Wright B, Duffy D, Linehan S, Kennedy HG. Psychiatric morbidity in male remanded and sentenced committals to Irish prisons. *Ir J Psychol Med* 2009; **26**: 169–73.

20. Daniel AE, Robins AJ, Reid JC, DE W. Lifetime and six-month prevalence of psychiatric disorders among sentenced female offenders. *Bull Am Acad Psychiatry Law* 1988; **16**: 333–42.

21. Denton B. Psychiatric morbidity and substance dependence among women prisoners: An Australian study. *Psychiatry Psychol Law* 1995; **2**: 173–7.

22. Dudeck M, Kopp D, Kuwert P, et al. Prevalence of psychiatric disorders in prisoners with a short imprisonment: Results from a prison in north Germany. *Psychiatr Prax* 2009; **36**: 219–24.

23. Duffy D, Linehan S, Kennedy HG. Psychiatric morbidity in the male sentenced Irish prisons population. *Ir J Psychol Med* 2006; **23**: 54–62.

24. Forry JB, Ashaba S, Rukundo GZ. Prevalence and associated factors of mental disorders among prisoners in Mbarara municipality, southwestern Uganda: A cross-sectional study. *BMC Psychiatry* 2019; **19**: 178.

25. Fovet T, Plancke L, Amariei A, et al. Mental disorders on admission to jail: A study of prevalence and a comparison with a community sample in the north of France. *Eur Psychiatry* 2020; **63**: e43.

26. Gunter TD, Arndt S, Wenman G, et al. Frequency of mental and addictive disorders among 320 men and women entering the Iowa prison system: Use-of the MINI-Plus. *J Am Acad Psychiatry Law* 2008; **36**: 27–34.

27. Herrman H, McGorry P, Mills J, Singh B. Hidden severe psychiatric morbidity in sentenced prisoners: An Australian study. *Am J Psychiatry* 1991; **148**: 236–9.

28. Indig D, Gear C, Wilhelm K. Comorbid substance use disorders and mental health disorders among New Zealand prisoners. Wellington, New Zeeland: New Zealand Department of Corrections, 2016.

29. Joshi P, Kukreja S, Desousa A, Shah N, Shrivastava A. Psychopathology and other contributing stressful factors in female offenders: An exploratory study. *Indian J Forensic Med Toxicol* 2014; **8**: 149–55.

30. Linehan SA, Duffy DM, Wright B, Curtin K, Monks S, Kennedy HG. Psychiatric morbidity in a cross-sectional sample of male remanded prisoners. *Ir J Psychol Med* 2005; **22**: 128–32.

31. López M, Saavedra FJ, López A, Laviana M. Prevalence of Mental Health problems in sentenced men in prisons from Andalucía (Spain). *Rev Esp Sanid Penit* 2016; **18**: 76–84.

32. Lukasiewicz M, Blecha L, Falissard B, et al. Dual diagnosis: prevalence, risk factors, and relationship with suicide risk in a nationwide sample of French prisoners. *Alcohol Clin Exp Res* 2009; **33**: 160–8.

33. Lukasiewicz M, Falissard B, Michel L, Neveu X, Reynaud M, Gasquet I. Prevalence and factors associated with alcohol and drug-related disorders in prison: a French national study. *Subst Abuse Treat Prev Policy* 2007; **2**: 1.

34. Lynch SM, Dehart DD, Belknap JE, et al. A multisite study of the prevalence of serious mental illness, PTSD, and substance use disorders of women in jail. *Psychiatr Serv* 2014; **65**: 670–4.

35. Mir J, Kastner S, Priebe S, Konrad N, Strohle A, Mundt AP. Treating substance abuse is not enough: comorbidities in consecutively admitted female prisoners. *Addict Behav* 2015; **46**: 25–30.

36. Mundt AP, Alvarado R, Fritsch R, et al. Prevalence Rates of Mental Disorders in Chilean Prisons. *PLoS ONE* 2013; **8:** e69109.

37. Mundt AP, Baranyi G. The Unhappy Mental Health Triad: Comorbid Severe Mental Illnesses, Personality Disorders, and Substance Use Disorders in Prison Populations. *Front Psychiatry* 2020; **11**: 804.

38. Nacher M, Ayhan G, Arnal R, et al. High prevalence rates for multiple psychiatric conditions among inmates at French Guiana's correctional facility: diagnostic and demographic factors associated with violent offending and previous incarceration. *BMC Psychiatry* 2018; **18**: 159.

39. Naidoo S, Mkize D. Prevalence of mental disorders in a prison population in Durban, South Africa. *Afr J Psychiatry* 2012; **15**: 30–5.

40. Nanéma D, Goumbri P, Karfo K, Ouango J, Ouédraogo A. Epidemiological and clinical aspects of mental disorders in prisons in Ouagadougou, Burkina Faso. *Annales Africaines de Psychiatrie* 2014; **3**: 59–75 (in French).

41. Piselli M, Attademo L, Garinella R, et al. Psychiatric needs of male prison inmates in Italy. *Int J Law Psychiatry* 2015; **41**: 82–8.

42. Piselli M, Elisei S, Murgia N, Quartesan R, Abram KM. Co-occurring psychiatric and substance use disorders among male detainees in Italy. *Int J Law Psychiatry* 2009; **32**: 101–7.

43. Proctor SL, Hoffmann NG, Raggio A. Prevalence of Substance Use Disorders and Psychiatric Conditions Among County Jail Inmates: Changes and Stability Over Time. *Crim Justice Behav* 2019; **46**: 24–41.

44. Schröder T. Psychische Erkrankungen bei männlichen Gefangenen im geschlossenen Vollzug. Lübeck, Germany: Universität zu Lübeck, 2005 (in German).

45. Simpson AIF, Brinded PMJ, Laidlaw TM, Fairley N, Malcolm F. The National Study of Psychiatric Morbidity in New Zealand Prisons - An Investigation of the Prevalence of Psychiatric Disorders among New Zealand Inmates. Wellington, New Zeeland: Department of Corrections, 1999.

46. Tye CS, Mullen PE. Mental disorders in female prisoners. *Aust N Z J Psychiatry* 2006; **40**: 266–71.

47. Vicens E, Tort V, Duenas RM, et al. The prevalence of mental disorders in Spanish prisons. *Crim Behav Ment Health* 2011; **21**: 321–32.

48. Widmann B. Die Prävalenz psychischer Störungen bei Frauen in Haft. Aachen, Germany: Rheinisch-Westfälischen Technischen Hochschule Aachen, 2006 (in German).

49. Wright B, Duffy D, Curtin K, Linehan S, Monks S, Kennedy HG. Psychiatric morbidity among women prisoners newly committed and amongst remanded and sentenced women in the Irish prison system. *Ir J Psychol Med* 2006; **23**: 47–53.

50. Zabala-Baños MC, Segura A, Maestre-Miquel C, et al. Mental disorder prevalence and associated risk factors in three prisons of Spain. *Rev Esp Sanid Penit* 2016; **18**: 13–23.

51. Zamzam R, Hatta SM. Specific Psychiatric Disorders Among Convicted Female Offenders in a Malaysian Prison. *Malaysian J Psychiatry* 2000; **8**: 34–42.

52. Zhong SL, Zhu XM, Chen YN, et al. High Psychiatric Morbidity and Comorbidity Among Female Prisoners in Hunan, China. *Front Psychiatry* 2020; **11**: 271.
